# HIV-1 Vpr Triggers Mitochondrial Destruction by Impairing Mfn2-Mediated ER-Mitochondria Interaction

**DOI:** 10.1371/journal.pone.0033657

**Published:** 2012-03-16

**Authors:** Chih-Yang Huang, Shu-Fen Chiang, Tze-Yi Lin, Shiow-Her Chiou, Kuan-Chih Chow

**Affiliations:** 1 Graduate Institute of Microbiology and Public Health, National Chung Hsing University, Taichung, Taiwan; 2 Department of Pathology, China Medical University Hospital, China Medical University, Taichung, Taiwan; 3 Graduate Institute of Biomedical Sciences, National Chung Hsing University, Taichung, Taiwan; University of Rochester, United States of America

## Abstract

Human immunodeficiency virus 1 (HIV-1) viral protein R (Vpr) has been shown to induce host cell death by increasing the permeability of mitochondrial outer membrane (MOM). The mechanism underlying the damage to the mitochondria by Vpr, however, is not clearly illustrated. In this study, Vpr that is introduced, via transient transfection or lentivirus infection, into the human embryonic kidney cell line HEK293, human CD4^+^ T lymphoblast cell line SupT1, or human primary CD4^+^ T cells serves as the model system to study the molecular mechanism of Vpr-mediated HIV-1 pathogenesis. The results show that Vpr injures MOM and causes a loss in membrane potential (MMP) by posttranscriptionally reducing the expression of mitofusin 2 (Mfn2) via VprBP-DDB1-CUL4A ubiquitin ligase complex, gradually weakening MOM, and increasing mitochondrial deformation. Vpr also markedly decreases cytoplasmic levels of dynamin-related protein 1 (DRP1) and increases bulging in mitochondria-associated membranes (MAM), the specific regions of endoplasmic reticulum (ER) which form physical contacts with the mitochondria. Overexpression of Mfn2 and DRP1 significantly decreased the loss of MMP and apoptotic cell death caused by Vpr. Furthermore, by employing time-lapse confocal fluorescence microscopy, we identify the transport of Vpr protein from the ER, via MAM to the mitochondria. Taken together, our results suggest that Vpr-mediated cellular damage may occur on an alternative protein transport pathway from the ER, via MAM to the mitochondria, which are modulated by Mfn2 and DRP1.

## Introduction

Human immunodeficiency virus type 1 (HIV-1) infection is characterized by a severe decrease in the number of CD4^+^ T lymphocytes, and several mechanisms have been proposed to explain it. One major factor associated with the reduction in CD4^+^ T lymphocytes is HIV-1 viral protein R (Vpr), a small 96-amino acid protein, that mediates cell cycle arrest, DNA damage, and apoptosis [1], [2], [3], [4], [5], [6], [7].

Several lines of evidence have suggested that the killing of cells by Vpr might be the result of targeting mitochondria through a direct interaction with adenine nucleotide translocator (ANT) on the mitochondrial inner membrane to permeabilize mitochondrial membrane and release cytochrome c [7], [8], [9], [10], [11]. However, silencing of ANT had no effect on Vpr-induced apoptosis whereas knockdown of Bax suppressed it [12]. In addition, Vpr was shown to form an ion channel *in vitro* via its hydrophobic segment (amino acids 55–83) [8], [11] penetrating phospholipid bilayers [8], [11], [13]. Moreover, the configuration of Vpr C-terminal transmembrane domain (TMD) is similar to that of certain viral proteins, such as Myxoma virus M11L protein, vaccinia virus F1L protein, Epstein-Barr virus BHRF-1 protein and hepatitis C virus core protein. The C-terminal hydrophobic segments of these viral proteins contain mitochondrial targeting sequences (MTS), which are homologous to tail-anchored proteins [11], [13], [14], [15], [16], [17], [18]. The major feature of these proteins is that they all have one membrane anchor at the C-terminus, comprising one helical hydrophobic domain of 12 to 24 amino acids followed by positively charged amino acids [19], [20], [21]. However, details of the mechanism underlying the transport of Vpr to mitochondria and the deteriorating effect of Vpr on mitochondria have yet to be determined.

In this study, we demonstrate that Vpr is present on the endoplasmic reticulum (ER), mitochondria-associated membranes (MAM), and the mitochondrial outer membrane (MOM), possibly via the integration of its C-terminal transmembrane domain. Vpr integration on mitochondria could lead to mitochondrial fragmentation and disruption of the integrity of the MOM, which might result in the discharge of mitochondrial membrane potential (MMP). Moreover, we found that this effect could be due to a Vpr-related reduction in the protein levels of mitochondrial fusion protein, mitofusin 2 (Mfn2) via VprBP-DDB1-CUL4A ubiquitin ligase complex. However, transport of Vpr to the mitochondria is independent of the translocase of mitochondrial outer membrane (TOM). Our results suggest that Vpr could be transported to mitochondria by an alternative protein transport pathway, from the ER via the MAM, requiring at least three proteins, dynamin-related protein 1 (DRP1), Mfn2, and ATPase family, AAA domain containing 3A (ATAD3A) [22]. Interruption of this putative pathway by Vpr, by means such as reducing the expression of Mfn2 and DRP1, has a profound influence on the morphology and function of the mitochondria.

## Results

### Vpr is integrated in the mitochondrial outer membrane (MOM) by the C-terminal transmembrane domain, independent of the translocase of mitochondrial outer membrane (TOM)

**Figure 1:**
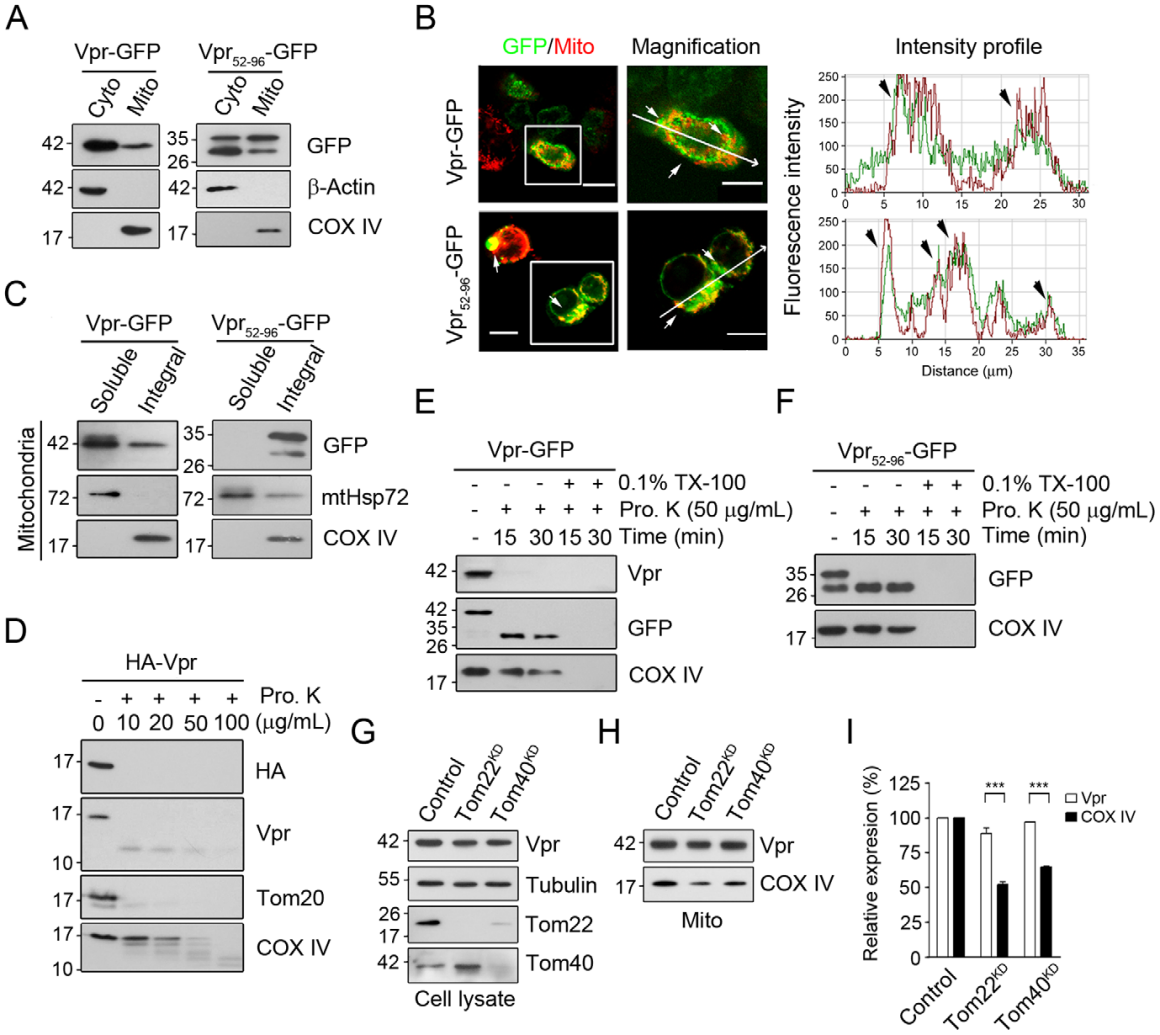
The C-terminal TMD is crucial to the integration of Vpr into MOM. **A**, The C-terminal TMD is required for the subcellular distribution of Vpr to mitochondria. **B**, Using confocal immunofluorescent microscopy, Vpr-GFP was localized on the mitochondria. Mitochondria were labeled using plasmid encoding mitochondria-targeted *Discosoma* red fluorescent protein (DsRed-Mito). Yellow fluorescence indicates the overlapping regions between the Vpr and mitochondria (white arrow). Bar: 10 µm. **C**, Treatment with 100 mM sodium carbonate (pH11.5) showed that Vpr and COX IV, as integral proteins of the mitochondrial inner membrane, were resistant to sodium carbonate, while the peripherally associated matrix protein mtHsp70 was separated in the soluble fraction. All experiments were independently repeated three times. **D**, Mitochondria isolated from HA-Vpr expressing cells were treated with proteinase K. The level of HA-Vpr was decreased after proteinase K treatment, indicating that the N-terminal HA was sensitive to protease. **E**, The C-terminal GFP fragment of the Vpr-GFP was resistant to proteinase K, indicating that the C-terminus of Vpr was protected. **F**, Resistance of the C-terminal GFP fragment of Vpr_52–96_-GFP to proteinase K treatment indicated that C-terminal GFP fragments were oriented toward the mitochondria. **G**, Following silencing of Tom22 or Tom40, cells were transfected with Vpr-GFP for 48 hours. Although expression of Tom22 or Tom40 was significantly reduced in gene silencing cells, expression of Vpr-GFP was not affected. **H**, The mitochondrial level of Vpr was not affected either in Tom22^KD^ or Tom40^KD^ cells as shown by Western blotting. **I**, The relative expression of mitochondrial Vpr-GFP in Tom22^KD^ and Tom40^KD^ cells was similar to that in control cells, although the mitochondrial protein COX IV was markedly reduced. The results are shown as the means ± S.D. of three independent experiments. ***The difference of relative expression is statistically significant (*p*<0.001) between the control and Tom22^KD^ cells or that and Tom40^KD^ cells.

Sequence analysis revealed that Vpr had a C-terminal transmembrane domain (TMD) followed by a positively charged amino acid, resembling the classical tail-anchored protein of ER and MOM [13], [16], [19], [21]. We therefore subcloned the C-terminal fragment (amino acid residues 52–96) of Vpr into a mammalian expression vector containing green fluorescent protein (GFP) to determine the localization of Vpr by subcellular fractionation and Western blotting. As shown in Figure 1A, Vpr-GFP proteins were detected in the mitochondria and in the cytosol. Fluorescence confocal microscopic images further confirmed that both Vpr- and Vpr_52–96_-GFP were present in the mitochondria (Fig. 1B). To identify the type of association, the mitochondria from Vpr-GFP expressing cells was isolated and treated with Na_2_CO_3_ to separate associated proteins from integral membrane proteins. As anticipated, Vpr-GFP and Vpr_52–96_-GFP were both identified in the pellet, containing membrane proteins, suggesting that Vpr is an integral membrane protein of mitochondria (Fig. 1C). To assess the orientation of Vpr integration, we used two types of constructs, one containing hemagglutinin at the amino-terminus (N-terminus), HA-Vpr, and the other containing GFP at the carboxy-terminus (C-terminus), Vpr-GFP or Vpr_52–96_-GFP, for a proteinase K protection assay. The N-terminal HA protein (Fig. 1D), but not the C-terminal fragment of either Vpr-GFP or Vpr_52–96_-GFP (Fig. 1E and 1F), was sensitive to proteinase K, suggesting that the N-terminus of Vpr was facing the cytosol while the C-terminus was anchored to the MOM. Previous studies have shown that importing mitochondrial protein is mediated by a mitochondrial protein translocation machinery, TOM complex, comprising the major receptors proteins, Tom20 and Tom22, and the central channel protein Tom40 [23]. Using Western blotting to examine mitochondrial content of ectopically expressed Vpr-GFP in Tom22 knockdown (Tom22^KD^) and Tom40^KD^ cells, we observed no significant difference in Vpr-GFP expression between Tom22^KD^ and the control cells or between Tom40^KD^ and the control cells (Fig. 1G). Similarly, silencing of Tom20 did not influence the mitochondrial import of Vpr (data not shown); however, mitochondria protein COX IV, which has been known to be imported into mitochondria via TOM complex, showed a marked decrease (∼0.5-fold) (Fig. 1H and 1I). These results suggest that the transport of Vpr to mitochondria is TOM-independent.

### Vpr protein is integrated into the endoplasmic reticulum (ER)

**Figure 2:**
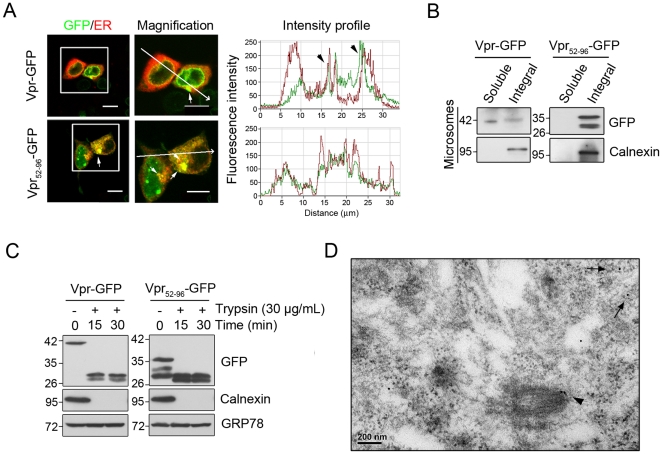
Transport of Vpr to the ER. **A**, Confocal immunofluorescent microscopy localized Vpr at the ER. ER was labeled using plasmid encoding ER-targeted *Discosoma* red fluorescent protein (DsRed-ER). Yellow fluorescence indicates the overlapped regions between Vpr and ER (white arrow). Bar: 10 µm. **B**, Treatment with 100 mM sodium carbonate confirmed that Vpr and the ER integral protein Calnexin were present in the alkaline-resistant fraction. **C**, Isolated microsomal fractions treated with trypsin indicate that Vpr inserted into the ER by the C-terminal TMD. **D**, Using immunogold electron microscopy, Vpr particles (15 nm) were shown to be located on the membrane of mitochondria (arrowhead) and ER (arrow). These results are representative of three independent experiments.

The presence of the C-terminal TMD sequence predicted that Vpr could be integrated into the ER as well. Using confocal fluorescence microscopy, we found that both Vpr-GFP and Vpr_52–96_-GFP were indeed located in the ER (Fig. 2A). Vpr was specifically associated with the ER, as shown by Na_2_CO_3_ washing of microsomal fractions (including the ER) to remove non-specific binding proteins (Fig. 2B). Using protease protection assay and Western blotting, we also found that Vpr in microsomal fractions was resistant to trypsin (Fig. 2C), indicating that, similar to what was observed in the mitochondria, Vpr was an integral protein of the ER. Furthermore, by employing immunogold transmission electron microscopy, Vpr proteins were identified on the MOM (Fig. 2D, arrowhead) and the ER (Fig. 2D, arrows). These results correspond well with our prior data. As a comparison, no immunogold signal (detecting Vpr) was detected in cells transfected with vectors only (data not shown). Statistically, 29.3% of the immunogold particles (detecting Vpr) were in the cytosol, 30.7% in the mitochondria, 18.7% in the vesicular structures and 9.3% in the ER.

### Vpr induces bulging of the mitochondria-associated membrane (MAM)

**Figure 3:**
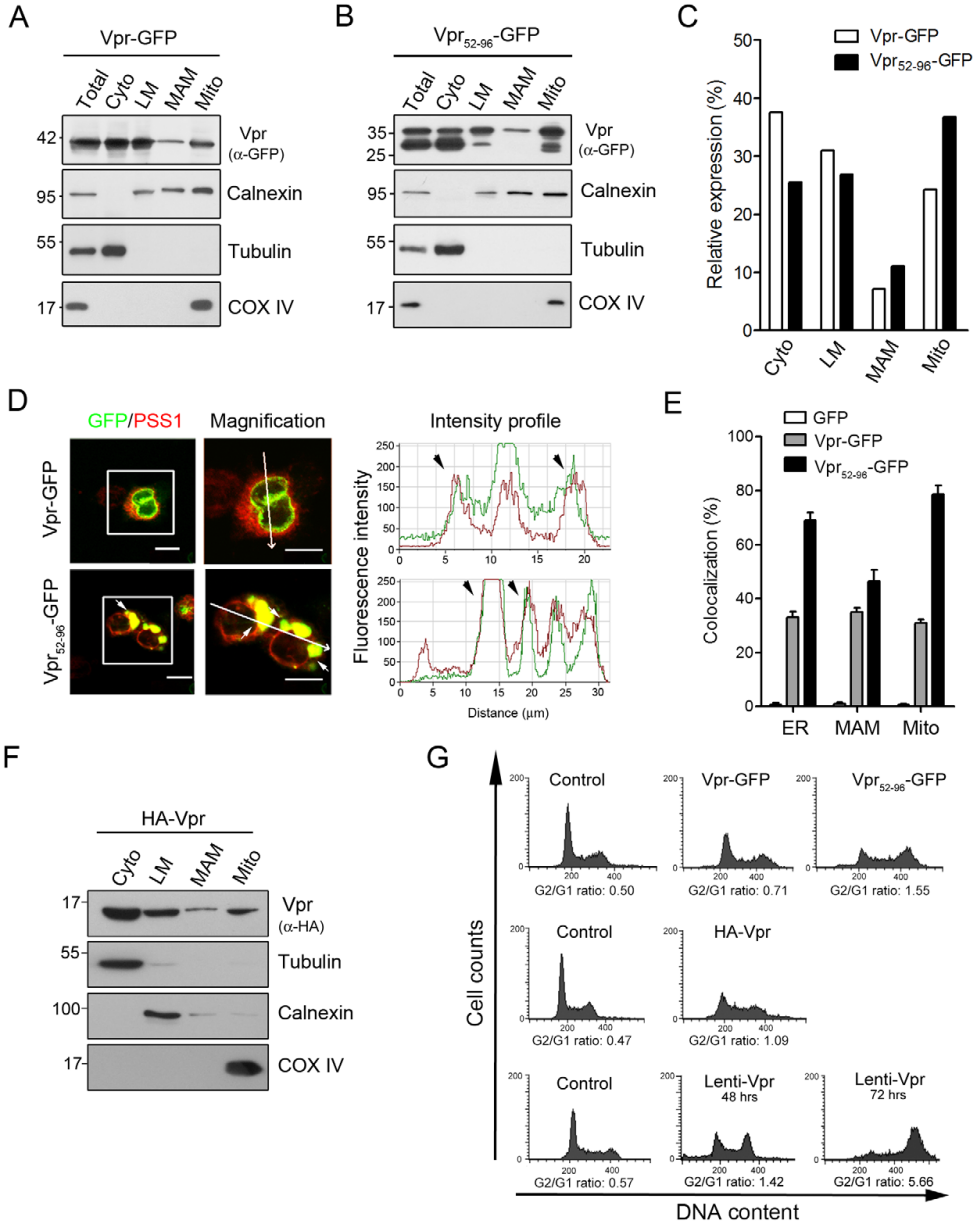
Vpr is presented in the MAM. **A**, Using Percoll self-generating gradient fractionation, Vpr-GFP was localized in the ER, MAM, and mitochondria. Cyto: cytosolic fraction, LM: microsomal fraction, Mito: mitochondrial fraction. **B**, Likewise, Vpr_52–96_-GFP was located in the ER, MAM, and mitochondria. **C**, Relative expression levels of Vpr-GFP and Vpr_52–96_-GFP in different subcellular fractions are similar. **D**, Immunofluorescent confocal microscopy showed that Vpr was co-localized with the MAM marker, PSS-1 protein. Bulged MAM became evident in Vpr_52–96_-GFP expressing cells. Yellow fluorescence (white arrow) indicates an overlap between Vpr (green fluorescence) and MAM (red fluorescence). Bar: 10 µm. **E**, Colocalization of Vpr_52–96_-GFP or Vpr-GFP with the marker of ER, MAM and mitochondria was assessed based on confocal images, and 100–120 cells were scored in three independent experiments. The results showed that both proteins were present in the three organelles. **F,** HEK293 cells were transfected with the plasmid encoding HA-Vpr for 48 hours. Cells were harvested and fractionated on a self-generated Percoll gradient. HA-Vpr was localized in the ER, MAM, and mitochondria. Cyto: cytosolic fraction, LM: microsomal fraction, Mito: mitochondrial fraction. **G,** HEK293 cells were either transfected with the plasmid encoding Vpr-GFP, Vpr_52–96_-GFP, or HA-Vpr for 48 hours, or infected with Lenti-Vpr (48 and 72 hours). Cells were harvested and cell cycle was analyzed by flow cytometry. Vpr-induced G2 arrest was observed in Vpr-expressing cells.

Detection of Vpr in both the ER and the mitochondria suggests that Vpr could be delivered separately to these two organelles following the synthesis of protein or transferred sequentially from the ER to the mitochondria [24], [25]. It is interesting to note that a connection between these two organelles and MAM has been hypothesized as a route of delivery for viral proteins from the ER to mitochondria, and it is possible that Vpr may be trafficked by this route. Using Percoll gradient and Western blotting, we found that Vpr-GFP and Vpr_52–96_-GFP were distributed in cytosol, ER, MAM, and purified mitochondria (Fig. 3A, 3B and 3C). Immunofluorescence confocal microscopic images verify that Vpr signals were co-localized with the marker signals of ER, MAM and mitochondria (Fig. 3D and 3E). Moreover, these images show that Vpr-GFP, and in particular Vpr_52–96_-GFP, were located mainly in the bulging ER/MAM, suggesting that overexpressed Vpr could be accumulated in the MAM, a prospective docking area for mitochondrial proteins prior to transportation [22].

To rule out what we observed above was due to GFP fusion, HA-Vpr expressing cells were fractionated and analyzed by Western blotting. Consistently, HA-Vpr was distributed in cytosol, ER, MAM, and purified mitochondria (Fig. 3F). Cell cycle analysis showed that Vpr-GFP, Vpr_52–96_-GFP, and HA-Vpr all led to an increase (relative to the control) in the G2/G1 ratio, which reflected its G2 arrest activity (Fig. 3G). In comparison, full-length Vpr was introduced into lentiviral expressing vector to mimic the function of native Vpr. Cell cycle analysis indicated that Lenti-Vpr infection caused a profound G2 arrest (Fig. 3G). Based on G2/G1 ratio, Vpr_52–96_-GFP (1.55) showed a comparable G2 arrest activity as Lenti-Vpr (1.42). On the other hand, HA-Vpr (1.09) and Vpr-GFP (0.71) induced lower G2/G1 ratios than Lenti-Vpr.

### Vpr expression reduces expression level of mitochondrial protein Mfn2

**Figure 4:**
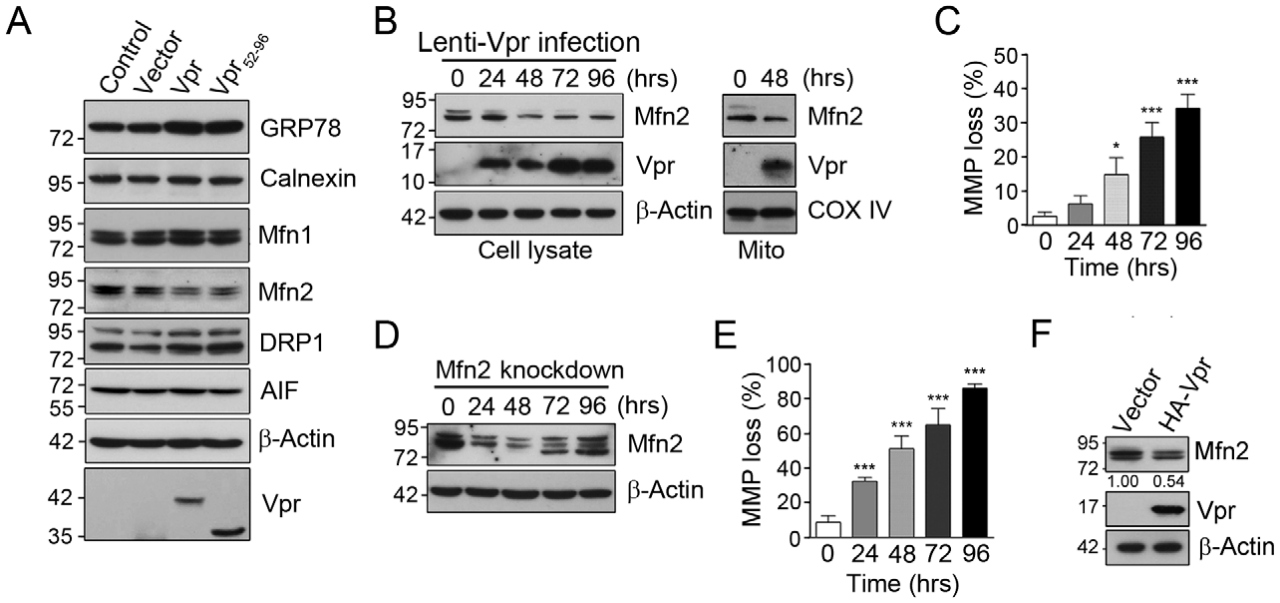
Vpr influences the expression level of GRP78 and Mfn2 proteins. **A**, At 48 hours post-transfection, expression of Vpr-GFP or Vpr_52–96_-GFP up-regulated GRP78 level, but reduced Mfn2 level in HEK293 cells. **B**, Vpr-related decrease in the expression of Mfn2 was time-dependent after Lenti-Vpr infection. **C**, Vpr-related loss of mitochondrial membrane potential (MMP) was also time-dependent, indicating that the effect of Vpr on MMP change was gradual, not immediate. **D**, Infection of HEK293 cells with lentivirus carrying siRNA to Mfn2 markedly reduced protein levels 48 hours after viral treatment. **E**, Similar to that mediated by Vpr, the Mfn2 silencing-induced loss of MMP was time-dependent. Results are the means ± S.D. of three independent experiments. For panels **C** and **E**, * (*p*<0.05) and *** (*p*<0.001) indicate significantly different from the control. **F,** HEK293 cells were transfected with the plasmid encoding HA-Vpr for 48 hours. Cells were harvested and analyzed by Western blotting. The expression of Mfn2 was reduced in HA-Vpr expressing cells.

Interestingly, following Vpr transfection or Lenti-Vpr infection, the expression level of Mfn2 was markedly reduced (Fig. 4A and 4B), while that of GRP78 was significantly increased (Fig. 4A). Vpr, however, did not influence the mRNA levels of GRP78 or Mfn2 (data not shown), suggesting that the influence of Vpr on these proteins could be post-transcriptional. It is worth noting that Vpr caused a loss in mitochondrial membrane potential (MMP) (Fig. 4C), a phenomenon that could also be induced by Mfn2 silencing (Fig. 4D and 4E). The influence of Vpr on MMP loss was approximately one third of that caused by Mfn2 silencing [26], [27], [28]. In addition, Mfn2 reduction was also observed in HA-Vpr expressing cells (Fig. 4F).

### Vpr protein induces abrasion of MOM and deformation of mitochondrial cristae

**Figure 5:**
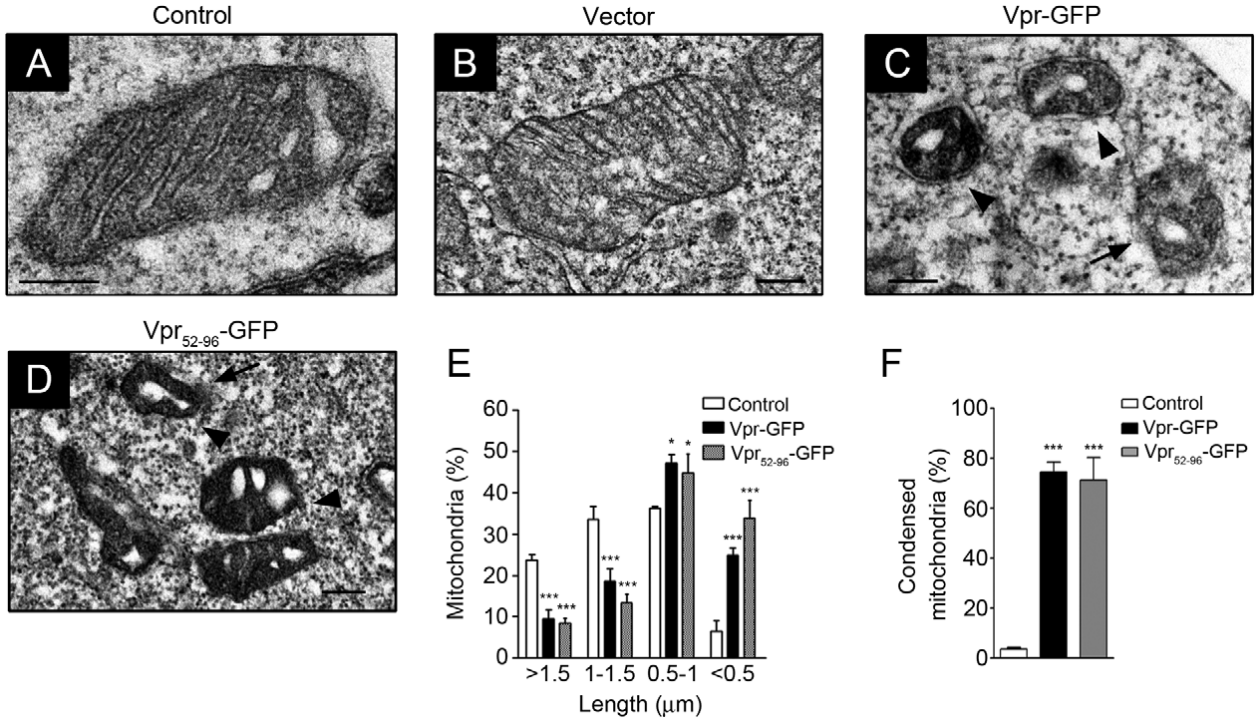
Vpr protein influences the morphology of mitochondria and integrity of MOM. **A**, Electron microscopic analysis of HEK293 cells showed that mitochondria were intact with a double-layer membrane and regular arrangement of cristae. Bar: 200 nm. **B**, Similar to wild-type cells, expression of GFP did not influence the morphology of the mitochondria. Bar: 200 nm. **C**, Following Vpr-GFP expression, marked changes in the architecture of the cristae was observed. Vpr led to swollen cristae, condensed matrix (arrowhead) and gradually disappearance of outer membrane (arrow). Bar: 200 nm. **D**, The same phenomenon was observed with Vpr_52–96_-GFP expressing cells. Mitochondria were condensed with swollen cristae. Bar: 200 nm. **E**, Based on TEM images, Vpr induced mitochondrial fragmentation and the length of mitochondria was evidently shorter. Twenty five cells were scored in three independent experiments with means ± S.D. **F**, Vpr also resulted in the condensation of mitochondria. For panels **E** and **F**, 25 cells were scored in three independent experiments with means ± S.D. *** indicates statistically significant difference (*p*<0.001) between the control and Vpr-expressing cells.

In a recent report we showed that in addition to inter-mitochondrial fusion, Mfn2 facilitates the fusion of the mitochondria and transport vesicles, which were budding off from the MAM, in an alternative transport pathway of mitochondrial proteins. This pathway requires at least three proteins, dynamin-related protein 1 (DRP1), the ATPase family AAA domain containing 3A (ATAD3A), and Mfn2 [22]. DRP1 and Mfn2 are GTPases, and ATAD3A is an ATPase. Deficiency in any of the three enzymes induces mitochondrial deformation, fragmentation, and cell death. Interestingly, compared with wild-type or GFP expressing cells (Fig. 5A and 5B), the morphology of the mitochondria was altered in Vpr-GFP and Vpr_52–96_-GFP expressing cells (Fig. 5C and 5D), as demonstrated by transmission electron microscopy. In these cells, the cristae were swollen, mitochondrial matrices condensed, and the outer membranes abraded (Fig. 5E and 5F).

### Knockdown of DRP1 (DRP1^KD^) expression induces the accumulation of Vpr in the MAM whereas actopic expression of Vpr induces nuclear translocation of DRP1

**Figure 6:**
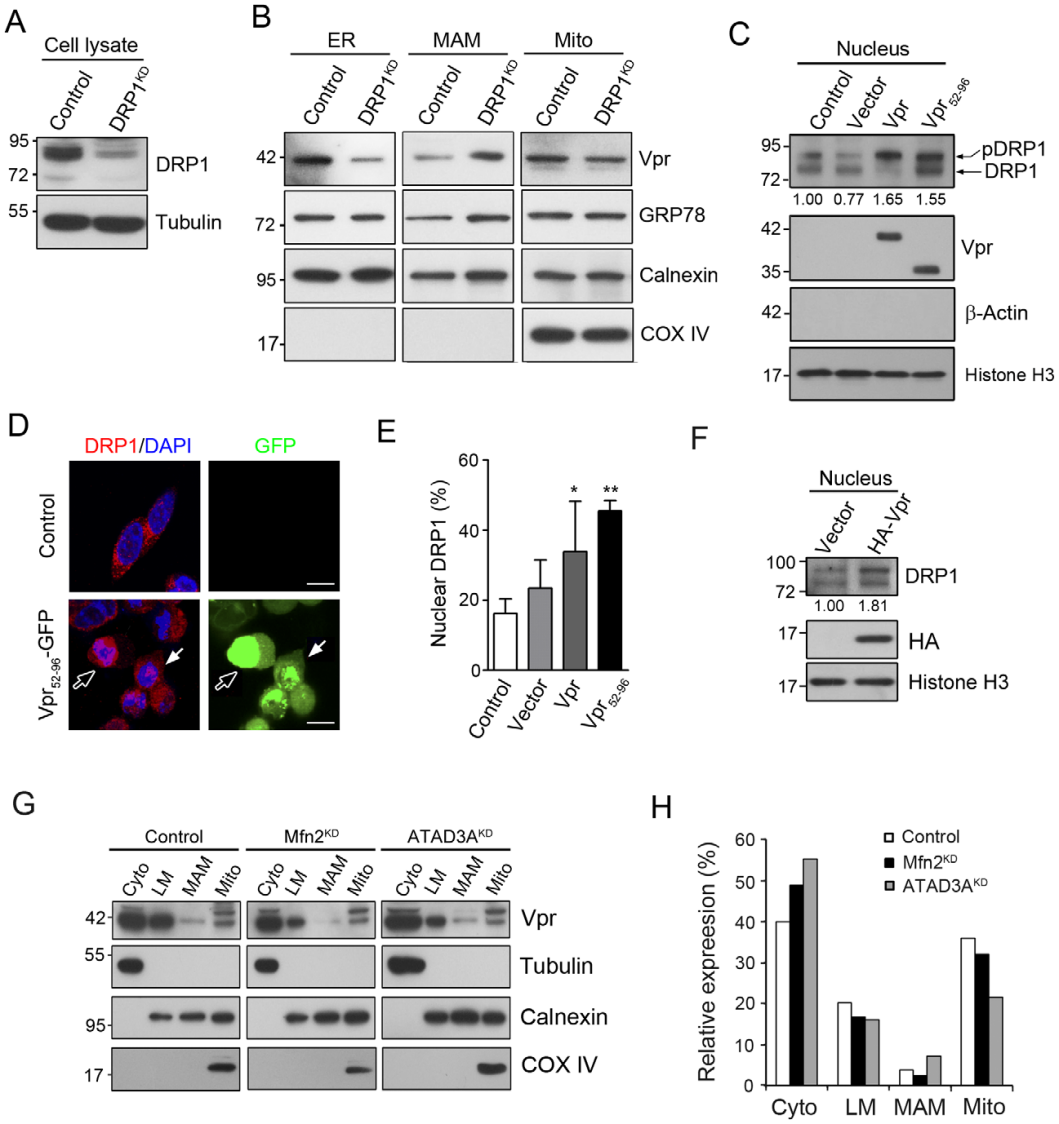
Knockdown of DRP1 (DRP1^KD^) expression induces the accumulation of Vpr in the MAM. Ectopic expression of Vpr, on the other hand, induces nuclear translocation of DRP1. **A**, Treatment with siRNA for 48 hours reduced the expression of DRP1 by approximately 80%. **B**, In DRP1^KD^ cells, protein levels of Vpr, Calnexin and GRP78 were increased in the MAM, while those of Vpr were decreased in the ER and mitochondria, indicating that the knockdown of DRP1 expression induced the accumulation of Vpr in the MAM. The results are representative of two independent experiments. **C**, The presence of Vpr or Vpr_52–96_ increased the levels of DRP1, in particular phosphorylated DRP1 (pDRP1), in the nucleus, as determined by Western blotting. These results are representative of two independent experiments. **D**, As shown by confocal immunofluorescence microscopy, ectopic expression of Vpr_52–96_ increased nuclear levels of Vpr and DRP1 (arrows with white margin). In some cells, both Vpr and DRP1 were co-localized in the nucleolus (white arrows). Bar: 10 mm. **E**, The increase of nuclear DRP1 in Vpr- or Vpr_52–96_-expressing cells. Fifty cells were scored in three independent experiments with means ± S.D. * (*p*<0.05) and ** (*p*<0.01) indicate significantly different from the control. **F,** HA-Vpr increased nuclear levels of DRP1. **G**, and **H**, The effect of gene knockdown of Mfn2 (Mfn2^KD^) or ATAD3A (ATAD3A^KD^) on the organelle distribution of Vpr was determined by Western blotting. Vpr level was increased in the cytosolic fractions, but it was reduced in the mitochondrial fractions in both Mfn2^KD^ and ATAD3A^KD^ cells. Band intensities were calculated using Image J. Relative intensities are shown at the bottom of each panel.

As noted above that ATAD3A, DRP1 and Mfn2 are involved in the importation of mitochondrial protein from the MAM, a prospective docking area prior to shipment [22]. Furthermore, Vpr could reduce Mfn2 expression. Interestingly, the knockdown of DRP1 (DRP1^KD^) (Fig. 6A), an up-stream GTPase which was essential for fission of transport vesicles from the MAM, altered the distribution of Vpr in the ER, MAM, and mitochondria: a reduction in the ER and mitochondria, but an increase in the MAM (Fig. 6B). Moreover, the ectopic expression of Vpr or Vpr_52–96_ increased the levels of nuclear DRP1, in particular the phosphorylated DRP1 (pDRP1) as determined by Western blotting (Fig. 6C). These results were confirmed by confocal immunofluorescence microscopy, in which Vpr_52–96_ increased the nuclear levels of Vpr and DRP1 (Fig. 6D). In some cells, DRP1 was co-localized with Vpr at the nucleolus. An increase in nuclear DRP1 was correlated with the expression of Vpr or Vpr_52–96_ in the cells. The differences were statistically significant (Fig. 6E). Consistently, HA-Vpr changed the distribution of DRP1 (Fig. 6F) as Vpr-GFP or Vpr_52–96_-GFP did. Knockdown of Mfn2 (Mfn2^KD^) or ATAD3A (ATAD3A^KD^) increased cytosolic Vpr levels, and reduced mitochondrial Vpr levels (Fig. 6G and 6H), which could be resulted from the accumulation of transport vesicles.

### Vpr-containing transport vesicles are formed from the ER/MAM and these vesicles fuse into mitochondria as determined by the time-lapse confocal fluorescence microscopy

**Figure 7:**
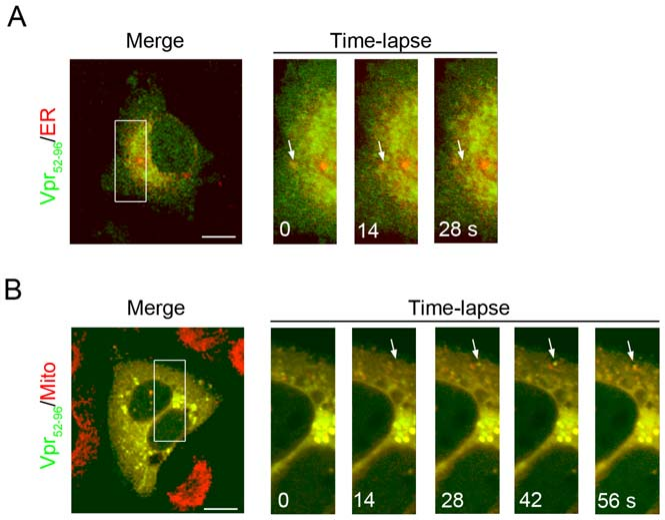
Transportation of Vpr protein from the ER/MAM to the mitochondria as determined by the time-lapse confocal fluorescence microscopy. **A**, Confocal fluorescence time-lapse images of Vpr_52–96_-GFP and CFP-ER co-expressed HEK293 cells show the formation of Vpr-containing transport vesicles (green fluorescence) from the ER/MAM (red fluorescence; yellow fluorescence indicates the merge of the green and the red fluorescence. Arrows indicate the region (ER/MAM, yellow fluorescence) where the Vpr_52–96_-GFP emerges. Intensity of yellow fluorescence in the ER/MAM decreases following the appearance of Vpr-containing transport vesicles (green fluorescence, T = 14 s–28 s). **B**, Fusion of the Vpr-containing transport vesicles (green fluorescence, Vpr_52–96-_GFP) into the mitochondria (red fluorescence, MitoTracker-Red) in real time. As shown in time-lapse series, Vpr-containing transport vesicle (green fluorescence) approached a mitochondrion (red fluorescence, T = 28 s), and then fused into the mitochondrion (T = 56 s). Arrows indicate Vpr_52–96_-GFP molecules that merge into the mitochondrion, and the fusion spot appears as yellow fluorescence (arrow). Results of the confocal fluorescence microscopy demonstrate that Vpr is budding off from the ER/MAM and forms within a spherical vesicle, which moves to the vicinity of the mitochondrion and then fuse into the organelle.

To record the formation of Vpr-containing transport vesicles from the ER/MAM and the fusion of these vesicles into the mitochondria in real time, we employed time-lapse confocal fluorescence microscopy. As shown in the time series of Fig. 7A, some of Vpr signals (green fluorescence) were overlapped with those of the ER/MAM (red fluorescence). The overlapped areas appeared as yellow fluorescence. Some other Vpr signals were found to be budding off from the side of the ER/MAM, and emerged as small green fluorescent vesicles (T = 14 s–28 s, Fig. 7A). Immediately, the Vpr-containing vesicles (green fluorescence, Fig. 7B) were found to approach a mitochondrion (T = 28 s, red fluorescence, Fig. 7B) and then fuse into the mitochondrion (T = 56 s, Fig. 7B). The new fusion site appeared as yellow fluorescent spot. Taken together, these data clearly indicated that Vpr could be budding off from ER/MAM and formed within a transport vesicle, which moved to the neighborhood of a mitochondrion and fused into the organelle. The rate of Vpr formation from the MAM was faster than that of transport vesicle fusion into the mitochondrion.

### Vpr-mediated mitochondrial damage causes cell death in HEK293 and CD4^+^ T cells under normal growth condition or serum starvation

**Figure 8:**
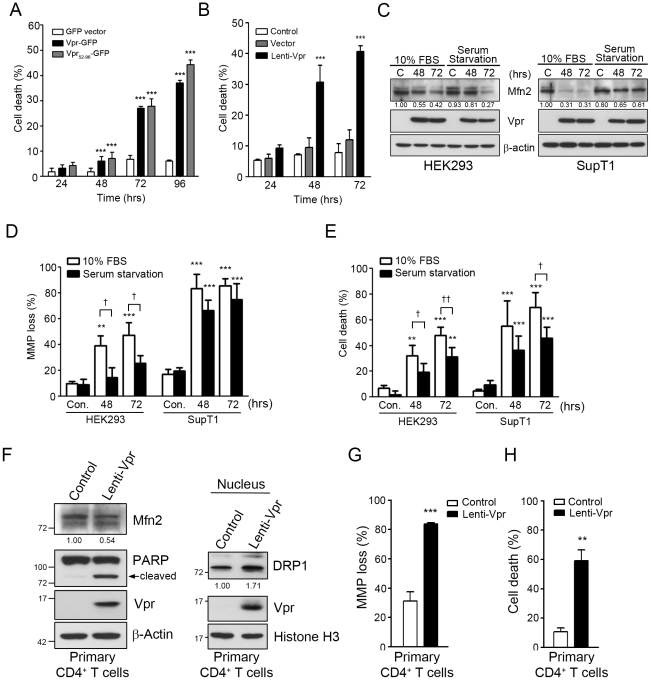
Vpr-mediated mitochondrial damage causes cell death in HEK293 and CD4^+^ T cells under normal growth condition or serum starvation. **A**, HEK293 cells were transfected with GFP vector the plasmid encoding Vpr-GFP or Vpr_52–96-_GFP, and harvested at different time (hours) post-transfection for PI staining. The percentage of dead cells among GFP-expressing cells was determined by flow cytometry. *** (*p*<0.001) indicates significantly different from the GFP vector control. **B**, HEK293 cells were infected with lenti-vector (control) or Lenti-Vpr and harvested at different time (hours) post-infection for PI staining. The percentage of dead cells was determined by flow cytometry. *** (*p*<0.001) indicates significantly different from the control. **C,** HEK293 and SupT1 cells were grown in 10% FBS or starved for 24 hours, and infected with Vpr-expressing lentivirus for 48 and 72 hours. The expression of Mfn2 was decreased in serum-starved HEK293 or SupT1 cells. The quantitative expression of Mfn2 was measured by Image J and normalized with the expression of β-actin. C indicates Vpr negative lentiviral control. **D,** MMP loss was determined after Vpr-expressing lentivirus infection. Vpr significantly impaired MMP in serum-starved HEK293 and SupT1 cells. **E,** Vpr expression led to cell death in serum-starved HEK293 and SupT1 cells. For panels **D** and **E**, results are the means ± S.D. of three independent experiments. * (*p*<0.05), ** (*p*<0.01) and ** (*p*<0.001) indicate significantly higher than the Vpr negative lentiviral control (Con.). † (p<0.05) and †† (*p*<0.01) indicate significantly different between 10% FBS and serum starvation. **F,** Human primary CD4^+^ T cells were isolated from peripheral blood mononuclear cell (PBMC) and infected with Vpr-expressing lentivirus for 72 hours. The expression of Mfn2 was decreased and the expression of nuclear DRP1 was increased in human primary CD4^+^ cells. The relative expression levels of Mfn2 and DRP1 were measured by Image J and normalized with the expression of β-actin. **G,** MMP loss was determined after Vpr-expressing lentivirus infection. Vpr led to a significant MMP loss in human primary CD4^+^ T cells. **H,** Vpr expression led to cell death in human primary CD4^+^ T cells. For panels **G** and **H**, results are the means ± S.D. of three independent experiments. ** (*p*<0.01) and *** (*p*<0.001) indicate significantly higher than control human primary CD4^+^ T cells. Band intensities were calculated using Image J. Relative intensities are shown at the bottom of each panel.

To confirm that Vpr-mediated cellular damage would result in cell death, we transfected HEK 293 cells with GFP vector (control) or the plasmid encoding Vpr-GFP or Vpr_52–96-_GFP, and examined the death rate by flow cytometry. Death rates of Vpr-GFP and Vpr_52–96-_GFP expressing cells were significantly higher (*p*<0.001) at 48, 72, and 96 hours post-transfection (Fig. 8A). As a comparison, we infected HEK293 cells with lentiviral vector carrying Vpr. The death rate of Lenti-Vpr-infected cells, as observed in Vpr-GFP and Vpr_52–96-_GFP expressing cells after transfection, increasing proportionally over time following infection, were significantly higher (*p*<0.001) than the control at 48, 72, and 96 hours post-infection (Fig. 8A, 8B).

We further investigated whether Vpr can cause similar cellular damage in non-dividing cells under serum starvation. HEK 293 cells were starved for 24 hours, and infected with Vpr-expressing lentivirus for 72 hours. Our results showed that, compared with the Vpr-negative control cells, we observed the reduced Mfn2, increased leakage of MMP and enhanced cell death (*p*<0.01) in serum-starved Vpr-expressing cells, with significantly lower impact (*p*<0.05 for less MMP loss and *p*<0.01 for decreased cell death) than that under normal growth condition (Fig. 8C, 8D and 8E).

Considering that CD4^+^ T cells are the primary target of HIV-1, we further examined the influence of Vpr on Sup T1, a human CD4^+^ T lymphoblast cell line. Similar to that observed in HEK293 cells, Vpr-expressing lentivirus caused Mfn2 reduction, MMP loss (*p*<0.001) and cell death (*p*<0.001) in SupT1 cells under both normal growth condition and serum starvation, with slightly decreased effects (*p*<0.05 for less cell death) in serum-starved cells (Fig. 8C, 8D and 8E). It is worth noting that Vpr effects on MMP loss and cell death were more profound in SupT1 cells than in HEK293 cells.

The effect of Vpr on human primary CD4^+^ T cells was further examined. As observed in CD4^+^ SupT1 and HEK293 cells, Vpr-expressing lentivirus reduced Mfn2 expression and increased the level of DRP1 in the nucleus, which correlated with MMP leakage (*p*<0.001) and cell death (*p*<0.01) in primary CD4^+^ T cells (Fig. 8F, 8G and 8H).

### Enforced expression of Mfn2 and DRP1 relieves Vpr-induced apoptosis

**Figure 9:**
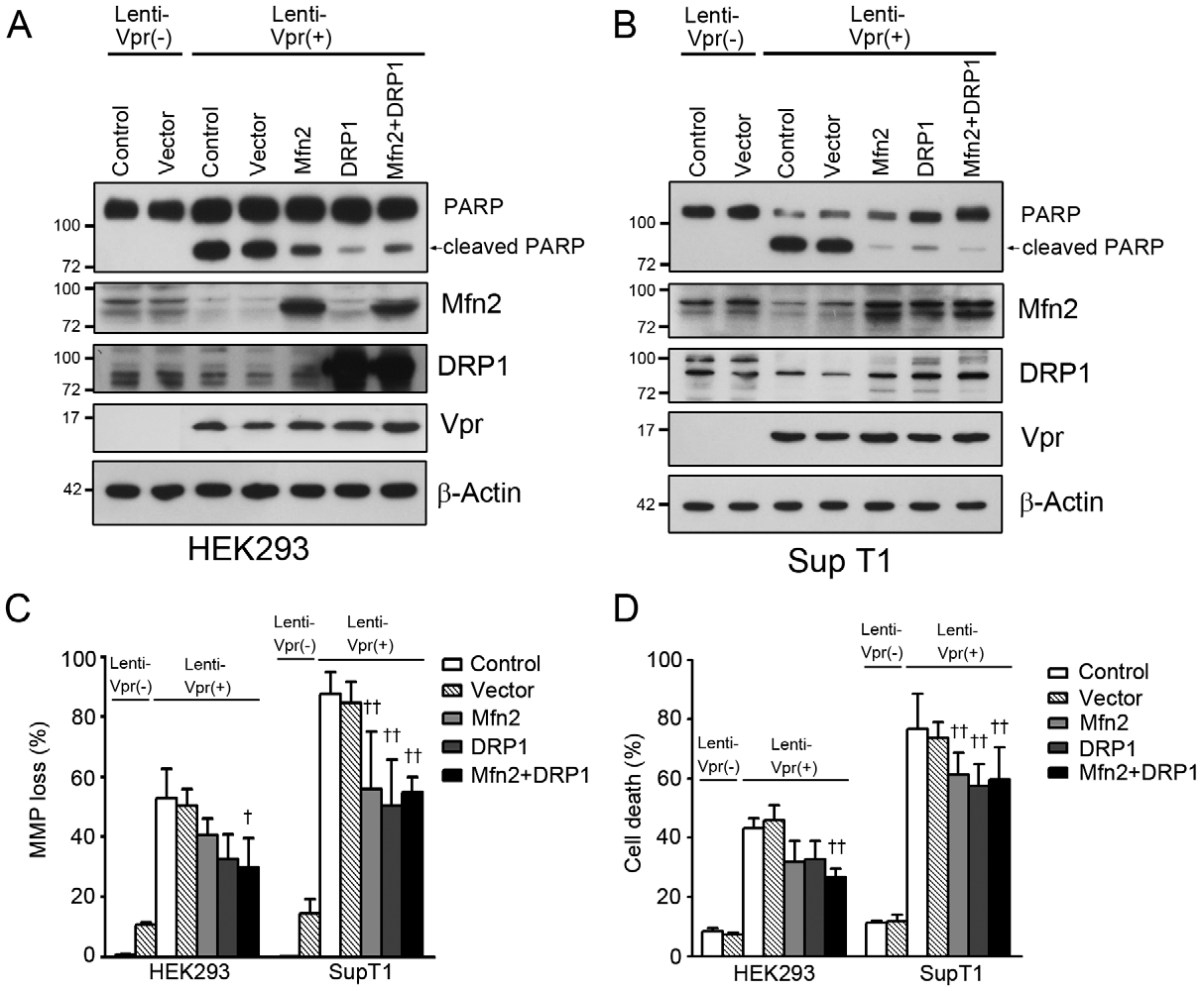
Enforced expression of Mfn2 and DRP1 relieves Vpr-induced apoptosis. After transient transfection of Mfn2, or DRP1 expression plasmid for 24 hours, cells were infected with Vpr-expressing lentivirus for 72 hours. **A,** In contrast to Lenti-Vpr negative (-) HEK293 cells (medium control and vector control), the 89 kDa cleavage fragment of PARP appeared exclusively in Lenti-Vpr positive (+) HEK293 cells. PARP cleavage was reduced in Mfn2-, DRP1- and Mfn2/DRP1-overexpressed Lenti-Vpr (+) HEK293 cells. **B,** PARP cleavage occurred only in Lenti-Vpr (+) SupT1 cells. The cleavage of PARP was reduced in Mfn2-, DRP1- and Mfn2/DRP1-expressed Lenti-Vpr (+) SupT1 cells. **C,** Overexpression of Mfn2, DRP1, and Mfn2/DRP1 decreased the percentage of MMP loss in Lenti-Vpr (+) HEK293 and SupT1 cells. **D,** Overexpression of Mfn2, DRP1, and Mfn2/DRP1 reduced Vpr-induced apoptosis. Results are the means ± S.D. of three independent experiments. For panels **C** and **D**, † (p<0.05) and †† (*p*<0.01) indicate significantly lower than Lenti-Vpr (+) cells pretreated with mock (medium control) or vector transfection.

As noted above that the reduction of Mfn2 protein and the decreased cytoplasmic distribution of DRP1 were the primary effects of Vpr, we therefore investigated whether Mfn2 and DRP1expression may relieve the cell death induced by Vpr. HEK 293 and SupT1 cells were respectively transfected with Mfn2, DRP1, and Mfn2/DRP1 expression plasmids for 24 hours, and infected with Vpr-expressing lentivirus for 72 hours. To evaluate the apoptotic cell death, we further examined the 89 kDa cleavage fragment of poly(ADP-ribose) polymerase (PARP), an apoptotic marker [29]. Vpr induced the apoptotic cleavage of PARP whereas the enforced expression of Mfn2 and DRP1 greatly reduced PARP cleavage in Vpr-positive HEK 293 cells (Fig. 9A). Similarly, overexpression of Mfn2 and DRP1 decreased the cleavage of PARP in Vpr-expressing SupT1 cells (Fig. 9B). Moreover, the losses of MMP and cell death induced by Vpr were also alleviated in Mfn2- and DRP1-expressing HEK 293 and SupT1 cells (Fig. 9C and 9D).

### Vpr down-regulated Mfn2 expression via VprBP-DDB1-CUL4A ubiquitin ligase in a proteasome-dependent manner

**Figure 10:**
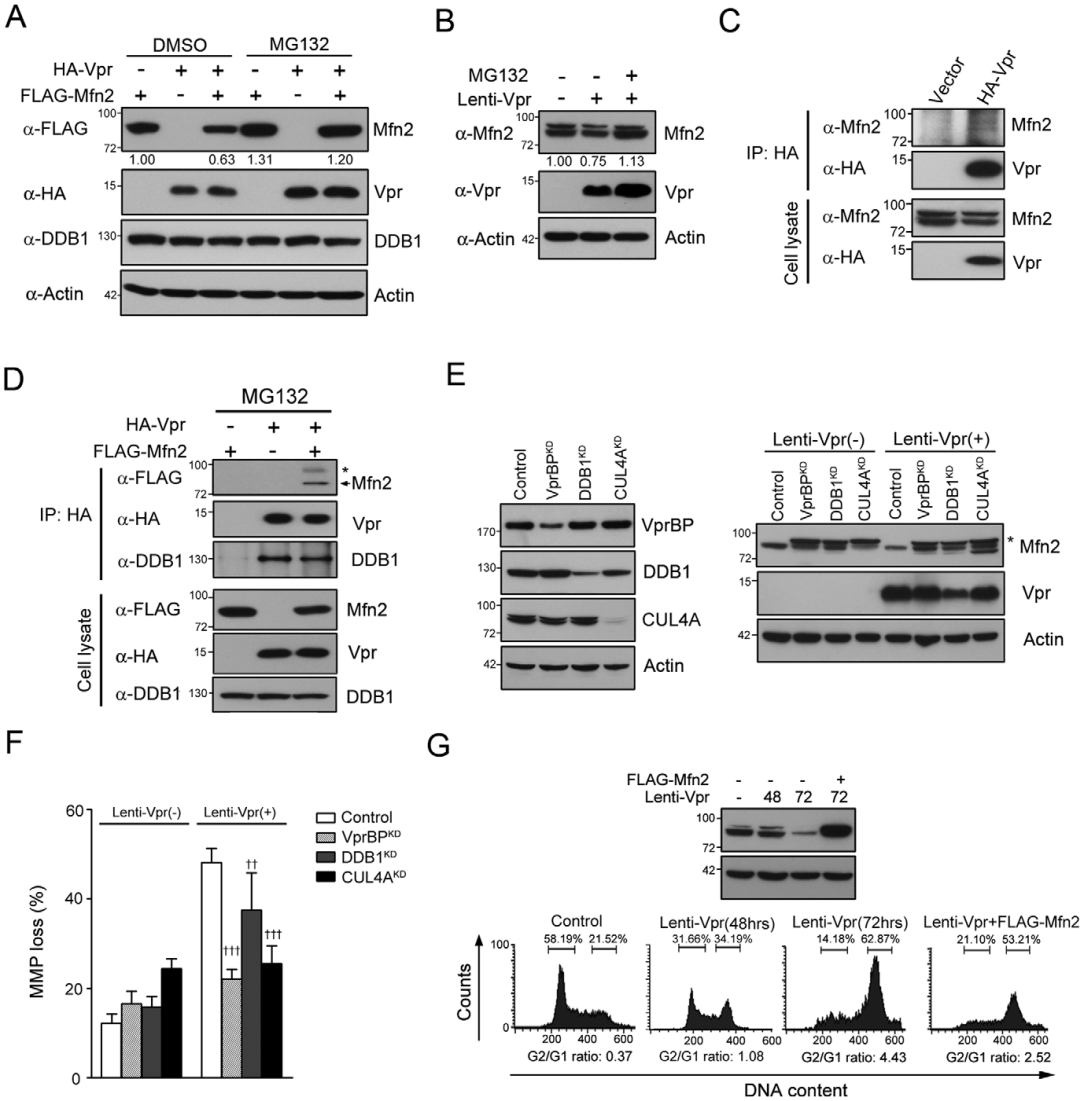
Vpr downregulated Mfn2 expression via VprBP-DDB1-CUL4A ubiquitin ligase complex. **A,** HEK293 cells were transfected respectively with plasmids encoding HA-Vpr and Flag-Mfn2 for 32 hours and treated with proteasome inhibitor MG132 (5 µM) for 16 hours. The expression of Mfn2 was recovered after MG132 treatment. **B,** HEK293 cells were infected with Lenti-Vpr for 56 hours and treated with proteasome inhibitor MG132 (5 µM) for 16 hours. The expression of Mfn2 was not decreased after MG132 treatment. **C**, HEK293 cells were transfected with the plasmid encoding HA-Vpr and harvested after 48 hrs. Cell lysates were immunoprecipitated with anti-HA antibody, and detected by Western blotting. **D,** HEK293 cells were transfected respectively with plasmids encoding HA-Vpr and Flag-Mfn2 for 32 hours and treated with MG132 (5 µM) for 16 hours. Cell lysates were immunoprecipitated with anti-HA antibodies, and the coimmunoprecipitated proteins were detected by Western blotting. * indicates the ubiquitinated Mfn2. **E,** HEK293 cells were silenced by shRNA against DDB1, VprBP or CUL4A, and infected with lentivirus carrying Vpr for 72 hours. The ubiquitinated Mfn2 was detected in VprBP^KD^, DDB1^KD^, CUL4A^KD^ cells. Moreover, Vpr infection did not decrease Mfn2 expression in VprBP^KD^, DDB1^KD^, CUL4A^KD^ cells. * indicates the ubiquitinated Mfn2. **F,** The MMP loss was determined by flow cytometry after Lenti-Vpr infection. Knockdown of DDB1, VprBP and CUL4A reduced the percentage of MMP loss after Lenti-Vpr infection. **G,** HEK293 cells were infected with Lenti-Vpr for 24 hours and transfected with the plasmid encoding FLAG-Mfn2 for 48 hours. Cells were harvested and analyzed by Western blotting and flow cytometry. Band intensities were calculated using Image J. Relative intensities are shown at the bottom of each panel.

Recent studies indicate that Vpr can act as an adaptor to interact with CUL4 E3 ubiquitin ligase complex, containing damaged DNA binding protein1 (DDB1), E3 ubiquitin ligase scaffold protein cullin 4A (CUL4A), and DDB1-CUL4A–associated factor 1 DCAF1 (also called VprBP) [30], [31], and down-regulate host cellular protein in a proteasome-dependent manner [31], [32], [33]. Because Vpr did not influence the mRNA expression of Mfn2 (data not shown), we examined whether the reduction of Mfn2 might be mediated by the VprBP-DDB1-CUL4A ubiquitin ligase complex. We measured the effect of proteasome inhibitor MG132 on Vpr-induced Mfn2 reduction to determine if the reduction of Mfn2 involved proteasomal degradation. After treatment with MG132, the protein level of Mfn2 and Vpr was increased in HA-Vpr and Flag-Mfn2 co-expressing cells (Fig. 10A). Moreover, the Mfn2 expression did not decrease in Lenti-Vpr-infected cells after MG132 treatment, suggesting that Vpr-induced reduction on Mfn2 might be mediated through proteasomal degradation (Fig. 10B).

A co-immunoprecipitation (co-IP) assay was performed on transfected cell lysates to examine whether Vpr was directly bound to Mfn2. In the co-IP assay, we found that there was weak interaction between Vpr and endogenous Mfn2 (Fig. 10C). However, Vpr was markedly coimmunoprecipitated with Mfn2 in MG132-treated transfected cells, indicating that the proteins were transiently presented as a complex in the cells (Fig. 10D). To explore whether Mfn2 degradation was regulated by Vpr-VprBP-DDB1-CUL4A ubiquitin complex, we silenced VprBP, DDB1, and CUL4A, and infected cells with Lenti-Vpr. The result showed that Mfn2 protein was not decreased in VprBP-, DDB1- and CUL4A-slienced Lenti-Vpr-infected cells. Interestingly, the band of ubiquitinated Mfn2 was increased in VprBP^KD^, DDB1^KD^, and CUL4A^KD^ Lenti-Vpr-infected cells, suggesting that Vpr-mediated Mfn2 reduction was via DDB1-CUL4A-VprBP ligase complex (Fig. 10E).

Because Vpr-induced Mfn2 reduction would lead to MMP loss, we measured the percentage of MMP loss in Vpr-expressing cells. Notably, compared with control cells, MMP loss was reduced in VprBP^KD^-, DDB1^KD^-, and CUL4A^KD^- cells after Lenti-Vpr infection, indicating that Vpr targeted Mfn2 to damage mitochondria through interacting with VprBP-DDB1-CUL4A ligase complex (Fig. 10F). In addition to maintaining mitochondria homeostasis, Mfn2 has been shown to inhibit cell proliferation by arresting cell cycle in the G0/G1 phases [34], [35], [36]. We transfected the plasmid encoding FLAG-Mfn2 into Lenti-Vpr-infected cells and analyzed the cell cycle by flow cytometry. Compared with Lenti-Vpr-infected cells, which were arrested at G2 phase (G2/G1 ratio: 4.43), enforced expression of Mfn2 indeed relieved G2 arrest (G2/G1 ratio: 2.52) (Fig. 10G). These results indicate that G2 arrest activity of Vpr may be attributed in part to its ability to down-regulated Mfn2 expression via VprBP-DDB1-CUL4A ubiquitin ligase in a proteasome-dependent manner.

## Discussion

The present study demonstrated that the major cytotoxic effect of HIV-1 Vpr might be damage to the mitochondria. This could be caused by the integration of C-terminal TMD on the mitochondrial outer membrane (MOM) reducing mitochondrial membrane potential (MMP), thereby leading to fragmentation of the mitochondria and swelling of the cristae. Moreover, Vpr markedly reduced the protein levels of Mfn2 via VprBP-DDB1-CUL4A ubiquitin ligase complex, in which gene deficiency is closely associated with mitochondrial dysfunction and cellular apoptosis.

An elegant study by Jacotot *et al.* [37] showed that exogenously added recombinant Vpr (rVpr) induced the rapid dissipation of mitochondrial membrane potential (MMP), as well as the release of apoptogenic proteins, such as cytochrome c and apoptosis inducing factor (AIF), from the mitochondria. They further found that the influence of rVpr was via interaction with permeability transition pore complex (PTPC) or its components, e.g., adenine nucleotide translocator (ANT) and voltage-dependent anion channel (VDAC), facilitating proapoptotic Bax-related membrane permeabilization. On the other hand, anti-apoptotic Bcl-2 and inhibitory reagents of PTPC, e.g., cyclosporine A and bongkrekic acid, inhibit the cytotoxic effects of Vpr on nuclear apoptosis. However, a study by Andersen *et al.* [12] showed that the silencing of ANT expression alone did not affect Vpr-associated apoptosis. On the contrary, they showed that the toxic effect of Vpr was on the MOM, probably on Bax-targeting proteins [38]. Interestingly, the presence of a potential C-terminal TMD suggests that Vpr is a tail-anchored protein, which could be located on the ER and the MOM [11], [14], [16], [39], [40], [41]. Our results of protease protection support their findings in which Vpr is an integral protein with the N-terminus facing the cytoplasm. A short 20 amino-acid C-terminal stretch in the ER lumen or mitochondrial intermembranous space, containing seven arginine residues, further implies that this arginine-rich region may interact specifically with C-terminal aspartates of VDAC, thereby interfering with its activity [42]. However, the role of VDAC in Vpr-related cell death remains to be determined.

Like Vpr, VDAC was also detected on both the mitochondria and the ER, particularly in areas resembling MAM [43], contact sites between the ER and mitochondria at which Mfn2 on both organelles tethered them together to coordinate Ca^2+^ flow [44]. It is possible that Vpr influences the function of VDAC on Ca^2+^ homeostasis in both organelles. In fact, the Bcl-2 family proteins, e.g., antiapoptotic Bcl-2, or proapoptotic Bax and Bak, the effectors of Vpr were also detected on both organelles modulating inter-organelle Ca^2+^ exchange as well as rheostatically regulating the initiation of apoptosis [45]. The critical issue is whether the newly synthesized proteins simultaneously target both organelles or if they are synthesized in one organelle and subsequently transported to the others. In this study, we have demonstrated by time-lapse confocal fluorescence microscopy that transport of Vpr protein is from the ER via MAM to the mitochondria. We noticed a faster rate of Vpr formation from the MAM than that of transport vesicle fusion into the mitochondrion. This implicates that the two events could be regulated by different enzymes, such as DRP1 and Mfn2.

Several studies have characterized the function of Vpr fusion proteins. An earlier study in 1995 by Di Marzio *et al.* [46] showed that N-terminal HA tagged Vpr (HA-Vpr) retained G2 arrest activity whereas C-terminal HA and luciferase (either N- or C-terminal end) tagged Vpr fusion proteins lost cell cycle arrest activity. Later on in 2003, Waldhuber *et al.* [47] demonstrated that, compared with the wild type Vpr or N-terminal His-tagged Vpr, Vpr fused with N-terminal GFP (Vpr-GFP) or C-terminal GFP (Vpr-GFP) caused an earlier (24 hours) yet transient (not sustained to 72 hours) G2 arrest activity. Both GFP-Vpr and Vpr-GFP, nevertheless, could induce apoptosis as efficient as wild type Vpr. They suggest that G2 arrest activity of Vpr is separable from its activity to induce apoptosis [47]. Similarly, Anderson *et al.* [12] observed a defective G2 arrest activity despite of an intact apoptosis-inducing activity in Vpr-GFP. In contrast, another study by Muthumani *et al.* [48] showed that Vpr-GFP is fully functional as the wild type Vpr, with equivalent activities in G2 arrest (48 hours after transfection), nuclear translocation, replication kinetic, and infectivity for CD4^+^ T cells, macrophages, and dendritic cells. In our study, we observed a normal G2 arrest activity in Vpr_52–96_-GFP expressing cells, with a similar G2/G1 ratio as Lenti-Vpr infected cells (1.55 vs. 1.42 at 48 hours). On the other hand, the lower G2/G1 ratio of HA-Vpr (1.09) and Vpr-GFP (0.71) expressing cells could be due to the lower transfection rate (as opposed to the higher infection rate) along with the lower G2 arrest activity. Overall, the disparity in G2 arrest activity among Vpr fusion proteins [12], [46], [47], [48] reflects that this functional domain is sensitive to modification with variability. GFP fusion of Vpr, in particular, may (partially or completely) or may not compromised G2 arrest activity, possibly depending on the conformation of each individual construct [12], [48]. In this study, both Vpr_52–96_-GFP (comparable activity) and Vpr-GFP (lower activity) were active in causing G2 arrest, and showed comparable ability as Lenti-Vpr to lead to Mfn2 reduction, increase in nuclear DRP1, MMP loss and cell death.

An elegant study by Borgne-Sanchez *et al.* [49] has shown that the recombinant Vpr activates depolarization of MMP in isolated mitochondria. In intact cells, rVpr induces bulging in some organelles in addition to the fragmentation of the mitochondria and abrasion of the MOM. Although considerable work has been focused on endocytosis, some bulging organelles did not match with endocytotic markers and the essence of these structures has not been characterized further. Our data suggests that the effects of Vpr on mitochondria could be caused by a Vpr-related reduction in Mfn2, a GTPase which is essential for the fusion and tethering of membranes [50], [51]. The loss of functional Mfn2 would certainly reduce the frequency of fusion among mitochondria [26], [27], [28], [52], [53] as well as that between transport vesicles and mitochondria. The later events could causally lead to a shortage in phospholipid supply from the ER/MAM to mitochondria, resulting in abrasion of the MOM [22] and depolarization of MMP.

Dynamin-related protein 1 (DRP1) is also required for mitochondrial protein transport [22]. The loss of functional DRP1 would interrupt the formation and budding-off of transport vesicles from the MAM, leading to accumulation of cargo proteins in MAM, and appearing as bulging cytostructures in confocal fluorescence microscopic images. Interestingly, Vpr, in particular Vpr_52–96_, induced organelle bulging as well, suggesting that Vpr could influence DRP1 activity. Overlapping Vpr images with the MAM marker, phosphatidylserine synthase 1 (PSS1), in bulging areas support such a concept. Unlike Mfn2, Vpr did not reduce total protein levels of DRP1. Our previous study showed that cell stress could increase nuclear levels of DRP1 [54]. Because DRP1 contains DNA binding motifs, it is possible that DRP1 is translocated to the nucleus to protect genomic DNA when cells have been exposed to the genotoxic stress, e.g., HIV-1 replication, which requires the Vpr-associated formation of pre-integration complex [55], [56]. Nuclear translocation of DRP1 could then reduce cytoplasmic DRP1, and induce DRP1 deficiency-related cellular responses.

Our results indicated that Vpr reduced the expression of Mfn2, enhanced MMP loss and led to cell death in both cycling cells and serum-starved non-dividing cells, with a decreased impact on serum-starved cells. Overexpression of Mfn2 and DRP1 significantly decreased the loss of MMP and reduced apoptotic cell death caused by Vpr. These results show that reduction of Mfn2 and nuclear translocation of DRP1, being the marked effects of Vpr-mediated cellular damage, can be rescued, in part, by the enforced expression of Mfn2 and DRP1. However, the fact that Vpr-induced MMP loss and cell death are only partly reduced by Mfn2 and DRP1 suggests that more factors may be involved in the process.

Previous studies have shown that Vpr could interact with CUL4A ubiquitin complex to degrade proteins, such as UNG and SMUG [32], [33]. On the other hand, the stability of Mfn2 has been suggested to be regulated by the ubiquitin/proteasome system [57]. In our study, we showed here that Vpr downregulated Mfn2 via VprBP- DDB1-CUL4A ubiquitin ligase complex in a proteasome-dependent manner. However, our study does not rule out that there could be other factors, such as Parkin and PINK1, participating in Vpr-induced Mfn2 reduction [58]. CUL4A ubiquitin complex has been shown to be involved in the Vpr-mediated G_2_ arrest [59], [60], [61], [62]. Other than functioning in mitochondrial fusion, Mfn2 can act as a cell proliferation suppressor to inhibit cell cycle transition from the G1 to S phase [34], [35]. In this study, we showed that enforced expression of Mfn2 relieved G2 arrest in Lenti-Vpr-infected cells. Taken altogether, by reducing the expression of Mfn2 via VprBP-DDB1-CUL4A ubiquitin ligase complex, Vpr may allow G1 cells to proceed through S phase and finally stop at G2 phase due to some other mechanism. For example, Li *et al.* [63] has suggested that Vpr mediates G2 arrest through an S phase dependent mechanism which involves Chk1 activation. Mfn2 reduction to facilitate an S phase may represent an earlier event triggered by Vpr, which is required for later events to lead to G2 arrest.

Vpr lacks the classical mitochondrial targeting sequence [16] and our data proves that the transport of Vpr to mitochondria is independent of TOM complex. Furthermore, we show that Vpr protein is transported from the ER/MAM to the mitochondria. Using organelle fractionation and Western blotting to study the intracellular distribution of cytomegalovirus UL37 proteins, Bozidis *et al*. [64] have demonstrated that UL37 is detected in the microsomes/ER, MAM and mitochondria. Using deletion mutants and confocal fluorescence microscopy, Williamson and Colberg-Poley [65] have further shown that the predominant UL37 isoforms, pUL37x1 (also known as vMIA) is first detected in the ER, and then successively in MAM, and mitochondria. Recently, we demonstrated that apoptosis-inducing factor (AIF), a mitochondrial protein located in the intermembranous space of mitochondria, was transported into mitochondria via ER and MAM [66]. These studies not only correspond well with our current data, but also suggest that protein transport from the ER, via the MAM to the mitochondria could be a general phenomenon for some endogenous as well as exogenous polypeptides. It should be noted, nevertheless, that other explanations are possible. For instance, nuclear Vpr could directly induce DNA damage while cytoplasmic Vpr interacts with anti-apoptotic mitochondrial protein HAX1 (HS1-associated protein X-1) [67], decreasing the function of high Ca^2+^ affinity SERCA2a (sarco/endoplasmic reticulum Ca^2+^ transport ATPase 2a) for Ca^2+^ influx to the ER [68]. It is conceivable that Vpr could collaborate with HIV-1 Nef protein to alter the activities of inositol-1,4,5-triphosphate receptor (PI_3_R) in Ca^2+^ efflux from the ER [69]. On the other hand, HIV-1 protease could directly degrade retinoic acid-inducible gene 1 (RIG-I) and inhibit the initiation of mitochondrial antiviral signaling (MAVS) and the subsequent activation of interferon regulatory factor 3 (IRF3) [70], [71]. Vpr could also influence the transport of MAVS to the mitochondria. In this manner, collaboration among viral proteins could simultaneously increase cytoplasmic Ca^2+^ level, thereby facilitating viral production, preventing innate immunity and inducing cell death.

Because only a small amount of Vpr is incorporated into virus particles, virion-associated Vpr may not be sufficient to induce dramatic mitochondrial deformation and lead to apoptosis. In fact, Vpr that is expressed in low level may have anti-apoptotic activity [72], [73]. On the other hand, previous studies have shown that Vpr led to apoptosis over 48 hours of infection by adenovirus-based vector [3], [74], revealing the profound effect of Vpr due to post-infection gene expression. Similarly, our results showed that loss of MMP and increased apoptotic cell death were significant over 24 hours of infection, suggesting that Vpr-induced apoptosis is resulted from Vpr gene expression after HIV-1 reverse transcription.

In conclusion, we show that Vpr is not directly transported to the mitochondria. Instead, it is synthesized in the ER, and transported to the mitochondria via MAM and transport vesicles. This process requires at least three critical proteins, including Mfn2, DRP1, and ATAD3A. Moreover, Vpr reduces the expression of the Mfn2, resulting in abrasion of the MOM, leading to fragmentation of the mitochondria, swelling of the cristae, loss of MMP, release of apoptogenic proteins, and the initiation of cell death.

## Materials and Methods

### Ethics statement

Antibodies were raised in mice in strict accordance with the Guidebook for the Care and Use of Laboratory Animals of the Council of Agriculture, Executive Yuan, Taiwan. The specific details of our protocol (IACUC approval number: 98–89) were reviewed and approved by the Institutional Animal Care and Use Committee (IACUC), National Chung Hsing University (IACUC of NCHU), Taiwan. All efforts were made to minimize suffering and to reduce the number of mice.

Human peripheral blood mononuclear cell were obtained from healthy volunteers with informed consent. The protocol (IRB approval 100-IRB-07) was reviewed and approved by the institutional review board (IRB), National Chung Hsing University (IRB of NCHU), Taiwan.

### Expression plasmids and gene construction

Plasmids pAS1B-HA-Vpr and pEGFP-N1-Vpr were obtained from Dr. S. Benichou [75]. The pcDNA3.1-Vpr was obtained from Dr. B. E. Sawaya [6]. The MAM marker construct, FH-PSS-1, was provided by Dr. O. Kuge [76]. The ER marker, DsRed-ER, was from Dr. D. J. Snyders. Plasmids pcDNA3.1-Flag-Mfn2 and pcDNA3.1-DRP1 were obtained from Dr. R. J. Youle. The mitochondrial marker, DsRed-Mito was provided by Dr. F. Peruzzi. To generate the truncated mutant of Vpr, we performed PCR using Vpr-GFP plasmid as the template. The primer sequences for the truncated Vpr was as follows: 5′-CTACTCGAG**ATG**ACTTGG GCAGGAGTGG AA -3′ (nts 5261–5279, *Xho* I site is underlined and the initiation codon is in bald-phase letters, NC_001802); and 5′-GCAAAGCTTGGATCTACTGGCTCCATTTCT-3′ (nts 5373–5393, *Hin*d III site is underlined). The amplified DNA fragment corresponded to the C-terminal sequence of amino acid 52–96 of Vpr. The amplified DNA was digested with *Xho* I and *Hin*d III, and the small DNA fragment was removed using the QIAquick PCR purification kit (QIAGEN, Duesseldorf, Germany). Vpr-specific inserts were subcloned into the pEGFP-N1 vector. For infection, full-length Vpr was amplified and subcloned into pLKO-AS3w vector (Academia Sinica, Taipei, Taiwan). The pLKO.1-lentivirus constructs carrying small hairpin RNA (shRNA) against Tom20, Tom22, Tom40, DRP1, DDB1, VprBP, CUL4A and Mfn2, were obtained from the National RNAi Core Facility, Academia Sinica, Taipei, Taiwan.

### Cell culture and transient transfections

Human embryonic kidney cell lines HEK293 and 293T (HEK293 stably expressing the SV40 large T-antigen) were obtained from ATCC (Manassas, VA, USA) and grown in Dulbecco modified Eagle medium (DMEM) supplemented with 10% fetal bovine serum (FBS), 4 mM glutamine, 100 U/ml penicillin and 100 µg/ml streptomycin. The cells were grown to 80% confluence on the day of transfection. The pEGFP-N1-Vpr encoding Vpr-GFP was transfected into target cells using jetPEI cationic polymer transfection reagent (Polyplus-transfection Inc., Illkirch, France).

Human CD4^+^ T lymphoblast cell line SupT1 was obtained from ATCC and grown in RPMI1640 medium supplemented with 10% FBS, 4 mM glutamine, 100 U/ml penicillin and 100 µg/ml streptomycin. Transient transfections of SupT1 cells were performed by using jetPEI transfection reagent. Briefly, 2.5×10^6^ cells were transfected with 4 µg of Mfn2 or DRP1 expression plasmids. After 24 hrs of transfection, the medium was replaced with medium containing Vpr lentiviral supernatant to carry out spinoculation [77].

Peripheral blood mononuclear cells were obtained from healthy donors, and CD4^+^ lymphocytes were purified using anti-CD4 magnetic beads (Dynal, Invitrogen, California, USA) according to the manufacturer's instructions. Primary CD4^+^ T cells were cultured in complete RPMI and infected with lentiviral supernatant carrying Vpr by centrifugal inoculation (spinoculation) as described previously [77]. After 72 hours, CD4^+^ T lymphocytes were harvested and analyzed.

### Lentivirus production and infection

Lentiviral vector carrying shRNA or full-length Vpr insert was prepared using a three-plasmid transfection method. Briefly, the pMD.G containing vesicular stomatitis virus glycoprotein, and pCMV-Δ 8.91carrying HIV-based packaging plasmid were co-transfected with lentiviral vector (control) or with the shRNA-, or Vpr-expressing lentiviral vector into 293T cells. The medium was changed to fresh DMEM containing 10 mg/ml BSA 24 hours post-transfection. The supernatant containing lentivirus was collected at 48 hour and 72 hour post-transfection, respectively. To infect HEK293 cells, lentiviral supernatant was added directly to cells in the presence of 8 µg/ml polybrene. SupT1 cells were infected with lentivirus by centrifugal inoculation (spinoculation) as described previously [77], and the cells were washed five times with medium. Following infection, the cells were selected using 1 µg/ml puromycin.

### Production of recombinant Vpr protein and antibody

Recombinant Vpr protein was expressed from the pGEX-4T-Vpr plasmid carrying GST protein at the N-terminus. GST-Vpr fusion protein was extracted and purified using Glutathione Sepharose 4B (GE Healthcare, Amersham, UK), and eluted with 10 mM reduced glutathione. Affinity-purified GST-Vpr was used to immunize BALB/c mice and the specificity of antibodies was determined by Western blotting. Monoclonal antibodies were produced using a hybridoma technique, and Vpr-specific antibodies were screened using ELISA and Western blotting methods as described previously [78].

### Subcellular fractionation

HEK293 cells transfected with the plasmid encoding Vpr-GFP were resuspended in sucrose homogenization medium (0.25 M sucrose, 10 mM HEPES, pH 7.4) containing protease inhibitors (Roche, Basel Schweiz, Switzerland), incubated on ice for 10 min, and subjected to glass Dounce homogenizer (Wheaton, Illinois, USA). Following the removal of the nuclei and unbroken cells through two rounds of centrifugation at 1,000×*g* for 10 min, the crude mitochondrial fraction was pelleted by centrifugation at 10,500×*g* for 10 min. The supernatant was a cytosolic fraction. The cytosolic and mitochondrial fractions were normalized for cell equivalents, and the distribution of Vpr- GFP fusion protein and marker proteins was assessed by Western blotting as described [78].

### Fractionation of MAM, mitochondria, and microsomes

MAM, mitochondria, and microsomes were isolated from Vpr-GFP expressing cells as previously described [64], [79], [80], [81]. Briefly, after Dounce and removal of the nuclei and unbroken cells, the supernatant was centrifuged at 10,500×*g* for 10 min to separate the crude microsomal fraction (containing microsomes and cytosol) from the crude mitochondrial fraction (containing MAM and mitochondria). The crude microsomal fraction (supernatant) was subjected to an ultracentrifugation at 100,000×*g* in a Beckman SW41 rotor for 60 min at 4°C to pellet the microsomes and ER (LM fraction), while the supernatant contained only cytosol. The crude mitochondrial fraction (pellet) was resuspended in 300 µl of ice-cold mannitol buffer A (0.25 M mannitol, 0.5 mM EGTA, 5 mM HEPES, pH 7.4) and layered on top of 10 ml of a 30% Percoll suspension in mannitol buffer B (0.225 M mannitol, 1 mM EGTA, 25 mM HEPES, pH 7.4). Mitochondria and MAM fractions were separated in self-generating Percoll gradients by ultracentrifugation at 95,000×*g* in a Beckman SW41 rotor for 65 min at 4°C. The mitochondrial fraction was diluted five times in mannitol buffer B and subjected to centrifugation at 6,300×*g* for 10 min at 4°C to pellet the purified mitochondria. The MAM fraction was briefly centrifuged at 6,300×*g* for 10 min at 4°C to remove the mitochondria, and the supernatant was further centrifuged at 100,000×*g* in a Beckman SW41 rotor for 30 min at 4°C to obtain the purified MAM (pellet). All fractions were resuspended in sucrose homogenization medium and analyzed by Western blotting.

### Protease accessibility assays

Crude mitochondrial fractions obtained from HA-Vpr or Vpr-GFP expressing cells were resuspended in SM buffer (0.25 M sucrose, 10 mM MOPS-KOH, pH 7.2) or SMTX-1 buffer (SM buffer containing 0.1% Triton X-100). Equal quantities of mitochondria were divided into aliquots and treated with protease as previously described [82], [83]. Briefly, proteinase K was added to final concentrations of 10, 20, 50 or 100 µg/ml. The samples were incubated on ice for 15 or 30 min. The reaction was stopped by the addition of 4 mM phenylmethylsulfonyl fluoride. The proteins were precipitated using acetone and centrifuged at 12,000 rpm for 30 min. The precipitated proteins were analyzed by Western blotting with anti-GFP antibodies (1∶3,000, Abcam, Cambridge, UK) or with antibodies to COX IV, a mitochondrial marker protein. The same procedure was used for trypsin digestion (30 µg/ml) and the proteolysis was stopped with cocktail protease inhibitors (Roche, Basel Schweiz, Swiss).

### Alkaline extraction experiment

LM fractions, containing enriched microsomes, and crude mitochondrial fractions were resuspended in freshly prepared ice-cold 0.1 M sodium carbonate (pH 11.5) at a final concentration of 500 µg of protein per ml of sodium carbonate. After incubation on ice for 20 min, the extracts were centrifuged at 130,000×*g* for 60 min. The pellet was solubilized in SDS sample buffer. The supernatant proteins were precipitated using acetone and centrifugation, and then solubilized in SDS sample buffer prior to Western blot analysis.

### Western blot analysis and co-immunoprecipitation

A total of 30 µg of total lysates or 10 µg of subcellular fractions were separated using SDS-polyacrylamide gel electrophoresis (SDS-PAGE) and analyzed by Western blotting. Blots were incubated with the respective primary antibodies, such as rabbit anti-GFP (1∶3,000, Abcam, Cambridge, UK), rabbit anti-PARP (1∶3,000, Cell signaling, Massachusetts, USA), rabbit anti-CUL4A (1∶1,000, Cell signaling, Massachusetts, USA), rabbit anti-DDB1 (1∶1,000, Cell signaling, Massachusetts, USA), rabbit anti-VprBP (1∶1,000, Novus Biologicals, Colorado, USA), mouse anti-HA tag (1∶1,000, Santa Cruz Biotechnology, California, USA), goat anti-FACL4 (1∶100, Santa Cruz Biotechnology, California, USA), mouse anti-calnexin (1∶3000, Abcam, Cambridge, UK) or mouse anti-COX IV (1∶1000, Invitrogen, California, USA). Protein signals were detected using horseradish peroxidase-conjugated secondary antibodies (1∶10,000, GE Healthcare, Amersham, UK) and Immobilon Western Chemiluminescent HRP Substrate (Millipore, Massachusetts, USA). The digital images were processed in Adobe Photoshop 7.0. Each blot was stripped using Restore Western Blot Stripping Buffer (Pierce, Illinois, USA) and incubated with other antibodies. The results were analyzed and quantified by Image J software (NIH, MD, USA).

### Measurement of mitochondrial membrane potential

Cells were stained with cell permeable, green fluorescent, lipophilic dye 3, 30-dihexyloxacarbocyanine iodide (DiOC6; Invitrogen, California, USA) as described [84], [85]. The dye was freshly prepared (50 nM in PBS) and added to the suspension (10^6^ cells/ml). After 20 min of incubation at 37°C in the dark, the samples were analyzed using a flow cytometer (FACS Calibur, BD, California, USA).

### Indirect immunofluorescence and confocal microscopy

Cells, which were co-transfected with organelle specific plasmids, DsRed2-ER, DsRed-Mito, FH-hPSS1 and Vpr-GFP for 24 hours, were reseeded onto slides. After 48 hours, the transfected cells were fixed with 4% paraformaldehyde for 15 min at room temperature and analyzed by confocal microscopy. FH-hPSS1 expressing cells were permeabilized with 0.1% Triton X-100 for 15 min at room temperature prior to staining with mouse anti-HA tag (1∶500, Santa Cruz Biotechnology, California, USA), and Alexa 546 rabbit anti-mouse IgG secondary antibodies (1∶300, Invitrogen, California, USA). Imaging was captured by using a Laser Scanning Confocal Microscope, LSM510 (Carl Zeiss, New York, USA). Images were processed using Adobe Photoshop. For the real time observation, time-lapse confocal fluorescence microscopy was employed with an Olympus FV-1000 confocal microscope (Tokyo, Japan) and the images were analyzed by using FV10-ASW 3.0 software (Tokyo, Japan).

### Electron microscopy

Cells were fixed *in situ* on culture dishes with 2.5% glutaraldehyde (Sigma, Missouri, USA) in 100 mM phosphate buffer (PB) (pH 7.2) at 4°C overnight. Cells were washed with PB before post-fixation with 1% osmium tetroxide in PB for 1 hour. After washing with distilled water, the cells were suspended in 2% agarose, and the agarose blocks were trimmed and dehydrated in a serial dilution of ethanol for 15 min each. The blocks were further dehydrated three time using 100% ethanol for 15 min each, and infiltrated with 100% ethanol/LR white (1∶1) mixture overnight. The blocks were changed to LR white (Agar Scientific Ltd., Essex, UK) for continuous infiltration at 4°C for 24 hours before being transferred to a capsule filled with LR white. LR white was polymerized and solidified at 60°C for 48 hours. The resin blocks were trimmed and cut using ultramicrotome (Leica Ultracut R, Vienna, Austria). Thin sections were transferred to 200 mesh copper grids, and stained with 2% uranyl acetate for 15 min, and 2.66% lead citrate (pH 12.0) for 15 min prior to observation with a JEM1400 electron microscope (JEOL USA, Inc., Massachusetts, USA) at 100–120 kV.

### Post-embedding immunogold procedure for electron microscopy

Vpr-GFP expressing cells were fixed using 4% paraformaldehyde and 0.1% glutaraldehyde in PBS buffer at 4°C for 18 hours and then permeabilized with 0.2% TritonX-100 in PBS for 1 hour. After being washed with PBS 3 times, the cells were embedded in 2% agarose gel and the block was cut into several pieces prior to dehydration. Following dehydration in a series of ethanol of escalated concentration, samples were embedded in LR White resin. Thin sections were cut using ultramicrotome and transferred to nickel grids. The non-specific binding sites on the thin sections were blocked with 50 mM glycine and 1% BSA in PBS for 15 min. The sections were incubated with rabbit anti-GFP polycolonal antibodies (1∶100) or mouse anti-Vpr monoclonal antibodies (1∶20) in incubation buffer (0.1% BSA and 15 mM NaN_3_ in PBS) for 18 hours at 4°C, respectively. The antibodies were removed by washing the grids with incubation buffer 6 times and the grids were further incubated with 15 nm gold-conjugated secondary antibodies (1∶50) (Aurion, Wageningen, Netherlands). Following removal of the non-binding antibodies through repeated washing of the grids with incubation buffer, the sections were post-fixed with 2% glutaraldehyde in PBS for 5 min and stained with uranyl acetate and lead citrate prior to observation with a JEM1400 electron microscope.

### Flow cytometric analysis of dead cells

HEK 293 cells were transfected with GFP vector, or the plasmid encoding Vpr-GFP or Vpr_52–96-_GFP, and harvested at different time (hrs) post-transfection. Cells were stained with propidium iodide (PI) for 5 to 10 min. The percentage of dead cells (positive stain for PI) among GFP-expressing cells was determined by using a flow cytometer (FACS Calibur, BD, California, USA).

## References

[1] Emerman M, Malim MH (1998). HIV-1 regulatory/accessory genes: keys to unraveling viral and host cell biology.. Science.

[2] Bukrinsky M, Adzhubei A (1999). Viral protein R of HIV-1.. Rev Med Virol.

[3] Muthumani K, Choo AY, Hwang DS, Chattergoon MA, Dayes NN (2003). Mechanism of HIV-1 viral protein R-induced apoptosis.. Biochem Biophys Res Commun.

[4] Andersen JL, Planelles V (2005). The role of Vpr in HIV-1 pathogenesis.. Curr HIV Res.

[5] Muthumani K, Choo AY, Premkumar A, Hwang DS, Thieu KP (2005). Human immunodeficiency virus type 1 (HIV-1) Vpr-regulated cell death: insights into mechanism.. Cell Death Differ.

[6] Siddiqui K, Del Valle L, Morellet N, Cui J, Ghafouri M (2008). Molecular mimicry in inducing DNA damage between HIV-1 Vpr and the anticancer agent, cisplatin.. Oncogene.

[7] Godet AN, Guergnon J, Croset A, Cayla X, Falanga PB (2010). PP2A1 binding, cell transducing and apoptotic properties of Vpr(77–92): a new functional domain of HIV-1 Vpr proteins.. PLoS One.

[8] Piller SC, Ewart GD, Premkumar A, Cox GB, Gage PW (1996). Vpr protein of human immunodeficiency virus type 1 forms cation-selective channels in planar lipid bilayers.. Proc Natl Acad Sci U S A.

[9] Macreadie IG, Thorburn DR, Kirby DM, Castelli LA, de Rozario NL (1997). HIV-1 protein Vpr causes gross mitochondrial dysfunction in the yeast Saccharomyces cerevisiae.. FEBS Lett.

[10] Ferri KF, Jacotot E, Blanco J, Este JA, Kroemer G (2000). Mitochondrial control of cell death induced by HIV-1-encoded proteins.. Ann N Y Acad Sci.

[11] Chen CP, Kremer C, Henklein P, Schubert U, Fink RH (2010). Modulating the activity of the channel-forming segment of Vpr protein from HIV-1.. Eur Biophys J.

[12] Andersen JL, DeHart JL, Zimmerman ES, Ardon O, Kim B (2006). HIV-1 Vpr-induced apoptosis is cell cycle dependent and requires Bax but not ANT.. PLoS Pathog.

[13] Wecker K, Morellet N, Bouaziz S, Roques BP (2002). NMR structure of the HIV-1 regulatory protein Vpr in H2O/trifluoroethanol. Comparison with the Vpr N-terminal (1–51) and C-terminal (52–96) domains.. Eur J Biochem.

[14] Schuler W, Wecker K, de Rocquigny H, Baudat Y, Sire J (1999). NMR structure of the (52–96) C-terminal domain of the HIV-1 regulatory protein Vpr: molecular insights into its biological functions.. J Mol Biol.

[15] Everett H, Barry M, Lee SF, Sun X, Graham K (2000). M11L: a novel mitochondria-localized protein of myxoma virus that blocks apoptosis of infected leukocytes.. J Exp Med.

[16] Boya P, Pauleau AL, Poncet D, Gonzalez-Polo RA, Zamzami N (2004). Viral proteins targeting mitochondria: controlling cell death.. Biochim Biophys Acta.

[17] Stewart TL, Wasilenko ST, Barry M (2005). Vaccinia virus F1L protein is a tail-anchored protein that functions at the mitochondria to inhibit apoptosis.. J Virol.

[18] Ohta A, Nishiyama Y (2011). Mitochondria and viruses.. Mitochondrion.

[19] Wattenberg B, Lithgow T (2001). Targeting of C-terminal (tail)-anchored proteins: understanding how cytoplasmic activities are anchored to intracellular membranes.. Traffic.

[20] Borgese N, Brambillasca S, Soffientini P, Yabal M, Makarow M (2003). Biogenesis of tail-anchored proteins.. Biochem Soc Trans.

[21] High S, Abell BM (2004). Tail-anchored protein biosynthesis at the endoplasmic reticulum: the same but different.. Biochem Soc Trans.

[22] Fang HY, Chang CL, Hsu SH, Huang CY, Chiang SF (2010). ATPase family AAA domain-containing 3A is a novel anti-apoptotic factor in lung adenocarcinoma cells.. J Cell Sci.

[23] Baker MJ, Frazier AE, Gulbis JM, Ryan MT (2007). Mitochondrial protein-import machinery: correlating structure with function.. Trends Cell Biol.

[24] Schinzel A, Kaufmann T, Borner C (2004). Bcl-2 family members: integrators of survival and death signals in physiology and pathology [corrected].. Biochim Biophys Acta.

[25] Bozidis P, Williamson CD, Wong DS, Colberg-Poley AM (2010). Trafficking of UL37 proteins into mitochondrion-associated membranes during permissive human cytomegalovirus infection.. J Virol.

[26] Bach D, Pich S, Soriano FX, Vega N, Baumgartner B (2003). Mitofusin-2 determines mitochondrial network architecture and mitochondrial metabolism. A novel regulatory mechanism altered in obesity.. J Biol Chem.

[27] Chen H, Detmer SA, Ewald AJ, Griffin EE, Fraser SE (2003). Mitofusins Mfn1 and Mfn2 coordinately regulate mitochondrial fusion and are essential for embryonic development.. J Cell Biol.

[28] Pich S, Bach D, Briones P, Liesa M, Camps M (2005). The Charcot-Marie-Tooth type 2A gene product, Mfn2, up-regulates fuel oxidation through expression of OXPHOS system.. Hum Mol Genet.

[29] Shah GM, Shah RG, Poirier GG (1996). Different cleavage pattern for poly(ADP-ribose) polymerase during necrosis and apoptosis in HL-60 cells.. Biochem Biophys Res Commun.

[30] Wen X, Duus KM, Friedrich TD, de Noronha CM (2007). The HIV1 protein Vpr acts to promote G2 cell cycle arrest by engaging a DDB1 and Cullin4A-containing ubiquitin ligase complex using VprBP/DCAF1 as an adaptor.. J Biol Chem.

[31] Casey L, Wen X, de Noronha CM (2010). The functions of the HIV1 protein Vpr and its action through the DCAF1.DDB1.Cullin4 ubiquitin ligase.. Cytokine.

[32] Schrofelbauer B, Yu Q, Zeitlin SG, Landau NR (2005). Human immunodeficiency virus type 1 Vpr induces the degradation of the UNG and SMUG uracil-DNA glycosylases.. J Virol.

[33] Ahn J, Vu T, Novince Z, Guerrero-Santoro J, Rapic-Otrin V (2010). HIV-1 Vpr loads uracil DNA glycosylase-2 onto DCAF1, a substrate recognition subunit of a cullin 4A-ring E3 ubiquitin ligase for proteasome-dependent degradation.. J Biol Chem.

[34] Chen KH, Guo X, Ma D, Guo Y, Li Q (2004). Dysregulation of HSG triggers vascular proliferative disorders.. Nat Cell Biol.

[35] Jin B, Fu G, Pan H, Cheng X, Zhou L (2010). Anti-tumour efficacy of mitofusin-2 in urinary bladder carcinoma.. Med Oncol.

[36] Wang W, Lu J, Zhu F, Wei J, Jia C (2010). Pro-apoptotic and anti-proliferative effects of mitofusin-2 via Bax signaling in hepatocellular carcinoma cells.. Med Oncol.

[37] Jacotot E, Ravagnan L, Loeffler M, Ferri KF, Vieira HL (2000). The HIV-1 viral protein R induces apoptosis via a direct effect on the mitochondrial permeability transition pore.. J Exp Med.

[38] Ott M, Norberg E, Zhivotovsky B, Orrenius S (2009). Mitochondrial targeting of tBid/Bax: a role for the TOM complex?. Cell Death Differ.

[39] Morellet N, Bouaziz S, Petitjean P, Roques BP (2003). NMR structure of the HIV-1 regulatory protein VPR.. J Mol Biol.

[40] Borgese N, Brambillasca S, Colombo S (2007). How tails guide tail-anchored proteins to their destinations.. Curr Opin Cell Biol.

[41] Rabu C, Schmid V, Schwappach B, High S (2009). Biogenesis of tail-anchored proteins: the beginning for the end?. J Cell Sci.

[42] Bayrhuber M, Meins T, Habeck M, Becker S, Giller K (2008). Structure of the human voltage-dependent anion channel.. Proc Natl Acad Sci U S A.

[43] Shoshan-Barmatz V, Zalk R, Gincel D, Vardi N (2004). Subcellular localization of VDAC in mitochondria and ER in the cerebellum.. Biochim Biophys Acta.

[44] de Brito OM, Scorrano L (2008). Mitofusin 2 tethers endoplasmic reticulum to mitochondria.. Nature.

[45] Scorrano L, Oakes SA, Opferman JT, Cheng EH, Sorcinelli MD (2003). BAX and BAK regulation of endoplasmic reticulum Ca2+: a control point for apoptosis.. Science.

[46] Di Marzio P, Choe S, Ebright M, Knoblauch R, Landau NR (1995). Mutational analysis of cell cycle arrest, nuclear localization and virion packaging of human immunodeficiency virus type 1 Vpr.. J Virol.

[47] Waldhuber MG, Bateson M, Tan J, Greenway AL, McPhee DA (2003). Studies with GFP-Vpr fusion proteins: induction of apoptosis but ablation of cell-cycle arrest despite nuclear membrane or nuclear localization.. Virology.

[48] Muthumani K, Montaner LJ, Ayyavoo V, Weiner DB (2000). Vpr-GFP virion particle identifies HIV-infected targets and preserves HIV-1Vpr function in macrophages and T-cells.. DNA Cell Biol.

[49] Borgne-Sanchez A, Dupont S, Langonne A, Baux L, Lecoeur H (2007). Targeted Vpr-derived peptides reach mitochondria to induce apoptosis of alphaVbeta3-expressing endothelial cells.. Cell Death Differ.

[50] Chen H, Chan DC (2005). Emerging functions of mammalian mitochondrial fusion and fission.. Hum Mol Genet.

[51] Scott I, Youle RJ (2010). Mitochondrial fission and fusion.. Essays Biochem.

[52] Karbowski M, Lee YJ, Gaume B, Jeong SY, Frank S (2002). Spatial and temporal association of Bax with mitochondrial fission sites, Drp1, and Mfn2 during apoptosis.. J Cell Biol.

[53] Neuspiel M, Zunino R, Gangaraju S, Rippstein P, McBride H (2005). Activated mitofusin 2 signals mitochondrial fusion, interferes with Bax activation, and reduces susceptibility to radical induced depolarization.. J Biol Chem.

[54] Chiang YY, Chen SL, Hsiao YT, Huang CH, Lin TY (2009). Nuclear expression of dynamin-related protein 1 in lung adenocarcinomas.. Mod Pathol.

[55] Popov S, Rexach M, Zybarth G, Reiling N, Lee MA (1998). Viral protein R regulates nuclear import of the HIV-1 pre-integration complex.. EMBO J.

[56] Pandey RC, Datta D, Mukerjee R, Srinivasan A, Mahalingam S (2009). HIV-1 Vpr: a closer look at the multifunctional protein from the structural perspective.. Curr HIV Res.

[57] Neutzner A, Benard G, Youle RJ, Karbowski M (2008). Role of the ubiquitin conjugation system in the maintenance of mitochondrial homeostasis.. Ann N Y Acad Sci.

[58] Whitworth AJ, Pallanck LJ (2009). The PINK1/Parkin pathway: a mitochondrial quality control system?. J Bioenerg Biomembr.

[59] Belzile JP, Duisit G, Rougeau N, Mercier J, Finzi A (2007). HIV-1 Vpr-mediated G2 arrest involves the DDB1-CUL4AVPRBP E3 ubiquitin ligase.. PLoS Pathog.

[60] Hrecka K, Gierszewska M, Srivastava S, Kozaczkiewicz L, Swanson SK (2007). Lentiviral Vpr usurps Cul4-DDB1[VprBP] E3 ubiquitin ligase to modulate cell cycle.. Proc Natl Acad Sci U S A.

[61] Le Rouzic E, Belaidouni N, Estrabaud E, Morel M, Rain JC (2007). HIV1 Vpr arrests the cell cycle by recruiting DCAF1/VprBP, a receptor of the Cul4-DDB1 ubiquitin ligase.. Cell Cycle.

[62] Tan L, Ehrlich E, Yu XF (2007). DDB1 and Cul4A are required for human immunodeficiency virus type 1 Vpr-induced G2 arrest.. J Virol.

[63] Li G, Park HU, Liang D, Zhao RY (2010). Cell cycle G2/M arrest through an S phase-dependent mechanism by HIV-1 viral protein R.. Retrovirology.

[64] Bozidis P, Williamson CD, Colberg-Poley AM (2008). Mitochondrial and secretory human cytomegalovirus UL37 proteins traffic into mitochondrion-associated membranes of human cells.. J Virol.

[65] Williamson CD, Colberg-Poley AM (2010). Intracellular sorting signals for sequential trafficking of human cytomegalovirus UL37 proteins to the endoplasmic reticulum and mitochondria.. J Virol.

[66] Chiang SF, Huang CY, Lin TY, Chiou SH, Chow KC (2012). An alternative import pathway of AIF to the mitochondria.. Int J Mol Med.

[67] Yedavalli VS, Shih HM, Chiang YP, Lu CY, Chang LY (2005). Human immunodeficiency virus type 1 Vpr interacts with antiapoptotic mitochondrial protein HAX-1.. J Virol.

[68] Vafiadaki E, Arvanitis DA, Pagakis SN, Papalouka V, Sanoudou D (2009). The anti-apoptotic protein HAX-1 interacts with SERCA2 and regulates its protein levels to promote cell survival.. Mol Biol Cell.

[69] Manninen A, Saksela K (2002). HIV-1 Nef interacts with inositol trisphosphate receptor to activate calcium signaling in T cells.. J Exp Med.

[70] Lei Y, Moore CB, Liesman RM, O'Connor BP, Bergstralh DT (2009). MAVS-mediated apoptosis and its inhibition by viral proteins.. PLoS One.

[71] Solis M, Nakhaei P, Jalalirad M, Lacoste J, Douville R (2011). RIG-I-mediated antiviral signaling is inhibited in HIV-1 infection by a protease-mediated sequestration of RIG-I.. J Virol.

[72] Conti L, Rainaldi G, Matarrese P, Varano B, Rivabene R (1998). The HIV-1 vpr protein acts as a negative regulator of apoptosis in a human lymphoblastoid T cell line: possible implications for the pathogenesis of AIDS.. J Exp Med.

[73] Fukumori T, Akari H, Iida S, Hata S, Kagawa S (1998). The HIV-1 Vpr displays strong anti-apoptotic activity.. FEBS Lett.

[74] Muthumani K, Zhang D, Hwang DS, Kudchodkar S, Dayes NS (2002). Adenovirus encoding HIV-1 Vpr activates caspase 9 and induces apoptotic cell death in both p53 positive and negative human tumor cell lines.. Oncogene.

[75] Jacquot G, Le Rouzic E, Maidou-Peindara P, Maizy M, Lefrere JJ (2009). Characterization of the molecular determinants of primary HIV-1 Vpr proteins: impact of the Q65R and R77Q substitutions on Vpr functions.. PLoS One.

[76] Tomohiro S, Kawaguti A, Kawabe Y, Kitada S, Kuge O (2009). Purification and characterization of human phosphatidylserine synthases 1 and 2.. Biochem J.

[77] O'Doherty U, Swiggard WJ, Malim MH (2000). Human immunodeficiency virus type 1 spinoculation enhances infection through virus binding.. J Virol.

[78] Chen JT, Huang CY, Chiang YY, Chen WH, Chiou SH (2008). HGF increases cisplatin resistance via down-regulation of AIF in lung cancer cells.. Am J Respir Cell Mol Biol.

[79] Vance JE (1990). Phospholipid synthesis in a membrane fraction associated with mitochondria.. J Biol Chem.

[80] Schwer B, Ren S, Pietschmann T, Kartenbeck J, Kaehlcke K (2004). Targeting of hepatitis C virus core protein to mitochondria through a novel C-terminal localization motif.. J Virol.

[81] Bozidis P, Williamson CD, Colberg-Poley AM (2007). Isolation of endoplasmic reticulum, mitochondria, and mitochondria-associated membrane fractions from transfected cells and from human cytomegalovirus-infected primary fibroblasts.. Curr Protoc Cell Biol Chapter.

[82] Morgan KS (2003). Characterization of cellular proteins. Current protocols in cell biology.

[83] Henderson MP, Billen LP, Kim PK, Andrews DW (2007). Cell-free analysis of tail-anchor protein targeting to membranes.. Methods.

[84] Korchak HM, Rich AM, Wilkenfeld C, Rutherford LE, Weissmann G (1982). A carbocyanine dye, DiOC6(3), acts as a mitochondrial probe in human neutrophils.. Biochem Biophys Res Commun.

[85] Wang D, Masutani H, Oka S, Tanaka T, Yamaguchi-Iwai Y (2006). Control of mitochondrial outer membrane permeabilization and Bcl-xL levels by thioredoxin 2 in DT40 cells.. J Biol Chem.

